# Landscape of the oncogenic role of fatty acid synthase in human tumors

**DOI:** 10.18632/aging.203730

**Published:** 2021-12-08

**Authors:** Xulei Huo, Lairong Song, Da Li, Ke Wang, Yali Wang, Feng Chen, Liwei Zhang, Liang Wang, Junting Zhang, Zhen Wu

**Affiliations:** 1 Department of Neurosurgery, Beijing Tiantan Hospital, Capital Medical University, Beijing, China; 2 China National Clinical Research Center for Neurological Diseases, Beijing, China; 3 Beijing Key Laboratory of Brain Tumor, Beijing, China; 4 Department of Neuro-Oncology, Cancer Center, Beijing Tiantan Hospital, Capital Medical University, Beijing, China

**Keywords:** fatty acid synthase, tumor, expression, prognosis, phosphorylation, methylation, tumor-infiltrating immune cell

## Abstract

Background: Identifying a unique and common regulatory pathway that drives tumorigenesis in cancers is crucial to foster the development of effective treatments. However, a systematic analysis of fatty acid synthase across pan-cancers has not been carried out.

Methods: We investigated the oncogenic roles of fatty acid synthase in 33 cancers based on the cancer genome atlas and gene expression omnibus.

Results: Fatty acid synthase is profoundly expressed in most cancers and is an important factor in predicting the outcome of cancer patients. Further, the level of S207 phosphorylation was found to be improved in several neoplasms (e.g., colon cancer). Fatty acid synthase expression is related to tumor-infiltrating immune cells in tumors (e.g., CD8+ T-cell infiltration level in cervical squamous cell carcinoma). Moreover, hormone receptor binding- and fatty acid metabolic process-associated pathways are involved in the functional mechanisms of fatty acid synthase.

Conclusions: This study provides a complete understanding of the oncogenic role of fatty acid synthase in human tumors.

## INTRODUCTION

The identification and portrayal of novel oncogenic genes are critical for gaining a more comprehensive understanding of the complicated course of tumorigenesis owing to the intricacy of this process. The Cancer Genome Atlas (TCGA) project and gene expression omnibus (GEO) dataset contain useful genomic information sets of various tumors [1–3] and can be employed to identify the potential oncogenic role of fatty acid synthase (FASN).

**Figure 1:**
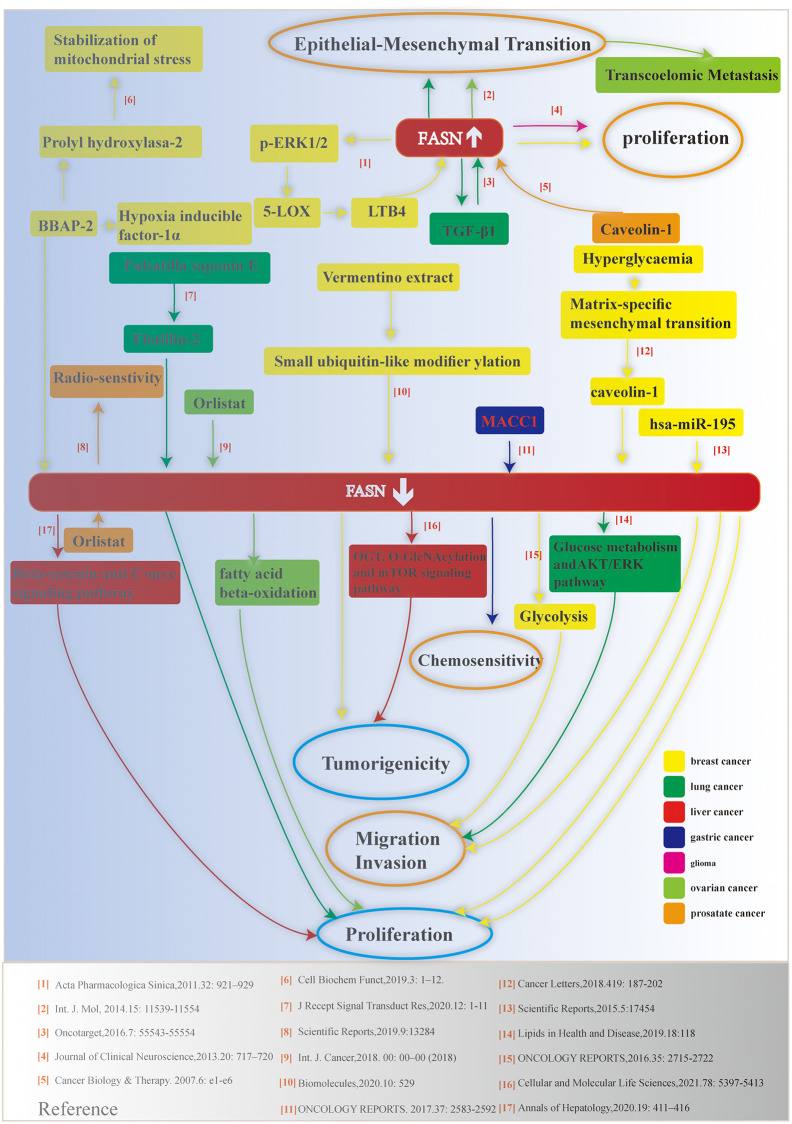
**Schematic depicting the relationship between FASN and various cancers**. Relevant references are indicated.

Endogenous fatty acid synthesis is catalyzed by FASN, a human lipogenic enzyme capable of *de novo* synthesis of fatty acids [4, 5]. The functional roles of FASN have been assessed in different species from the perspectives of physiology and pathology [6–9]. The human FASN protein is composed of six catalytic units. Starting from the N-terminus, these units include β-ketoacyl synthase (KS), acetyl/malonyl transacylase (AT/MT), β-hydroxyacyl dehydratase (DH), enoyl reductase (ER), β-ketoacyl reductase (KR), acyl carrier protein (ACP), and thioesterase (TE). The relationship between FASN and tumorigenesis of bladder cancer [10], meningioma [11], and breast cancer [12, 13] has been reported. In this study, we summarized laboratory-based results from cell or animal experiments with regarding to the relationship between FASN and various malignancies (Figure 1). Of note, no correlation was found between FASN and pan-cancer.

For our investigation, we utilized TCGA project and GEO datasets. Additionally, several features (e.g., gene expression, survival prognosis, genetic alteration, DNA methylation, protein phosphorylation, immune cells, or relevant cellular pathways) were gathered to evaluate the regulatory mechanism of FASN in the tumorigenesis of cancers.

## MATERIALS AND METHODS

### Gene mapping and protein structure analysis

Based on the UCSC genome browser on human Dec. 2013 (GRCh38/hg38) assembly [14], information related to the genome location of the *FASN* gene was obtained. Further, the “HomoloGene” function of the NCBI was used to conduct conserved functional domain analysis of the FASN protein in different species. The phylogenetic status across diverse types of species were evaluated with the “COBALT” function of NCBI.

### Gene expression analysis of TIMER2

We obtained the difference in FASN expression using the “Gene_DE” module of tumor immune estimation resource, version 2 (TIMER2). In tumors without contrast tissues or enough contrast group, the Gene Expression Profiling Interactive Analysis, version 2 (GEPIA2) web server [15] was utilized from the Genotype-Tissue Expression (GTEx) (*P* = 0.01, log2 fold change = 1).

We evaluated violin plots of FASN in various pathological stages across tumors using the “Pathological Stage Plot” module (GEPIA2). The log2 [TPM (Transcripts per million) +1] transformed expression results with a log-scale test were employed in the plots.

The Clinical Proteomic Tumor Analysis Consortium module [16] was used to determine the expression level of the total protein or phosphoprotein (S207, S724, S725, T976, S1174, S1411, S2198, and T2204 sites) of FASN (NP_004595.4) between cancers and corresponding tissue, respectively. Six tumors were screened: breast cancer, ovarian cancer, colon cancer, clear cell renal cell carcinoma (RCC), uterine corpus endometrial carcinoma (UCEC), and lung adenocarcinoma (LUAD).

### Gene expression analysis using human protein atlas

The Human Protein Atlas (HPA) database was utilized to obtain the expression data of FASN in different cells, tissues, and plasma. The data for plasma samples were estimated via mass spectrometry-based proteomics in the HPA database. “Low specificity” was defined by “Normalized expression ≥1 in at least one tissue/cell type, but not elevated in any tissue/cell type.”

Immunohistochemistry (IHC) images of FASN in five pairs of normal and tumor tissues, including breast invasive carcinoma (BRCA), cervical squamous cell carcinoma and endocervical adenocarcinoma (CESC), colon adenocarcinoma (COAD), liver hepatocellular carcinoma (LIHC), and prostate adenocarcinoma (PRAD) were downloaded from the HPA and analyzed.

### Gene expression analysis using Oncomine

We collected the different expression results of FASN between the tumor group and the corresponding normal group (*P* = 0.05, fold change = 1.5). Pooling analysis was performed across five comparisons. The median rank for FASN across each of the analyses, the *P*-value for the median-ranked analysis, and the legends of the enrolled studies were obtained.

### Survival prognosis analysis

We utilized the Kaplan-Meier “Survival Map” module (GEPIA2) [15] to obtain the overall survival (OS) and disease-free survival (DFS) map of FASN. The expression threshold for splitting the high/low expression groups was 50%. The Kaplan-Meier survival analysis module of GEPIA2 was used to obtain survival plots using the log-rank test.

The interface of the Kaplan-Meier plotter was used to pool the different GEO datasets for a series of analyses of OS, distant metastasis-free survival (DMFS), relapse-free survival (RFS), post-progression survival (PPS), first progression (FP), disease-specific survival (DSS), and progression-free survival (PFS). The cases of lung, ovarian, lung, gastric, and liver cancers were split into two groups by setting “autoselect best cutoff.” The hazard ratio (HR), 95% confidence intervals, and log-rank *P*-value were calculated, and Kaplan-Meier survival plots were generated. Clinical factors (e.g., histology, sex, smoking history, or chemotherapy) were also selected for a series of subgroup analyses.

Based on STATA 15.1 software (StataCorp LLC, College Station, TX, USA), we used the Kaplan-Meier function to perform a meta-analysis to pool the survival data of FASN.

### Genetic alteration analysis

We collected the genetic alteration features, alteration frequency, mutation type, copy number alteration (CNA), mutated site information, and three-dimensional (3D) structure from the cBioPortal web [17, 18]. The OS, DFS, and PFS for the tumors with or without FASN genetic alterations were collected. Log-rank *P*-value was obtained and Kaplan-Meier analysis was performed.

### Correlation of FASN and tumor mutational burden (TMB)/microsatellite instability (MSI)

TMB and MSI were obtained from the article, The Immune Landscape of Cancer and the Landscape of Microsatellite Instability Across 39 Cancer Types. The rank sum test detected two sets of data (*p* = 0.05). Spearman correlation was used to compare TMB/MSI and FASN gene expression.

### DNA methylation analysis

We utilized the MEXPRESS web with the query “FASN” to obtain the DNA methylation status of FASN of various probes (e.g., cg06234966, cg24715260) in glioblastoma (GBM). The beta value of each sample, and the Benjamini-Hochberg-adjusted *P*-value and Pearson correlation coefficient (R) values were obtained.

We used the GSE50923 dataset [19] to assess the methylation status in 54 GBM tissues and 24 normal brain tissues. Briefly, the “minfi” R package and boxplot were employed to perform normalization. The mean methylation level of FASN and the normalized beta value of each sample at the selected methylation probes (cg03386722 and cg23244421) were visualized using the ggviolin function of the ggpubr package, with the setting, stat_compare_means (paired = T, method = “wilcox.test”).

### Phosphorylation feature prediction

We used the open-access PhosphoNET database to obtain the predicted phosphorylation features of the S207, S724, S725, T976, S1174, S1411, S2198, and T2204 sites by searching the protein name “FASN.”

### Immune infiltration analysis

The TIMER2 web server was used to explore the association between FASN expression and immune infiltrates. Immune cells of CD8^+^ T-cells, CD4^+^ T-cells, cancer-associated fibroblasts, and NK cells were screened. Purity-adjusted Spearman’s rank correlation test was used to obtain *P*-values and partial correlation (cor) values.

### FASN-related gene enrichment analysis

The STRING website was utilized to obtain FASN-binding proteins, as demonstrated by experiments. The low confidence score was set as 0.150. Further, maximum number of interactors ≤50, physical interaction, and evidence were the basis of the interaction types.

The top 100 FASN-correlated targeting genes were obtained using the “Similar Gene Detection” module (GEPIA2). The pairwise gene correlation of FASN and the selected genes was obtained using the “correlation analysis” module. Moreover, a heatmap of the screened genes was obtained with the “Gene_Corr” module. Purity-adjusted Spearman’s rank correlation test was used to obtain *P*-values and partial correlation (cor) values.

We conducted Kyoto Encyclopedia of Genes and Genomes (KEGG) pathway analysis with the two sets of data. Database for Annotation, Visualization, and Integrated Discovery (DAVID) was utilized to acquire the functional annotation results. The enriched pathways were finally visualized with the “tidyr” and “ggplot2” R packages. Gene ontology (GO) enrichment analysis was also conducted with the “clusterProfiler" R package (Two-tailed *P* < 0.05).

### Data availability

The datasets analyzed in this study can be found in the online dataset. Requests for further access to the dataset can be directed to hxl950513@126.com.

### Highlights

A pan-cancer analysis of FASN. FASN is differentially associated with the prognosis of different tumor cases. The link between FASN and CD8^+^ T-cells, CD4^+^ T-cells, cancer-associated fibroblast infiltration, and NK cells. Enhanced phosphorylation level of S207 in several tumors, such as colon cancer. Hormone receptor binding- and fatty acid metabolic process-associated issues are involved in the cancerous role of FASN.

## RESULTS

### Gene mapping and protein structure analysis

In this study, we investigated the oncogenic function of FASN (NM_004104 or NP_004595.4, Supplementary Figure 1A). The protein structure of FASN is conserved among various species (e.g., *H. sapiens*, *G. gallus*, *C. elegans*) and generally involves the cond_enzymes (cl09938) domain and acyl_transf_1 (cl08282) domain (Supplementary Figure 1B). A phylogenetic tree (Supplementary Figure 2) revealed the evolutionary relationship of the FASN protein across various species.

### Gene expression analysis

**Figure 2:**
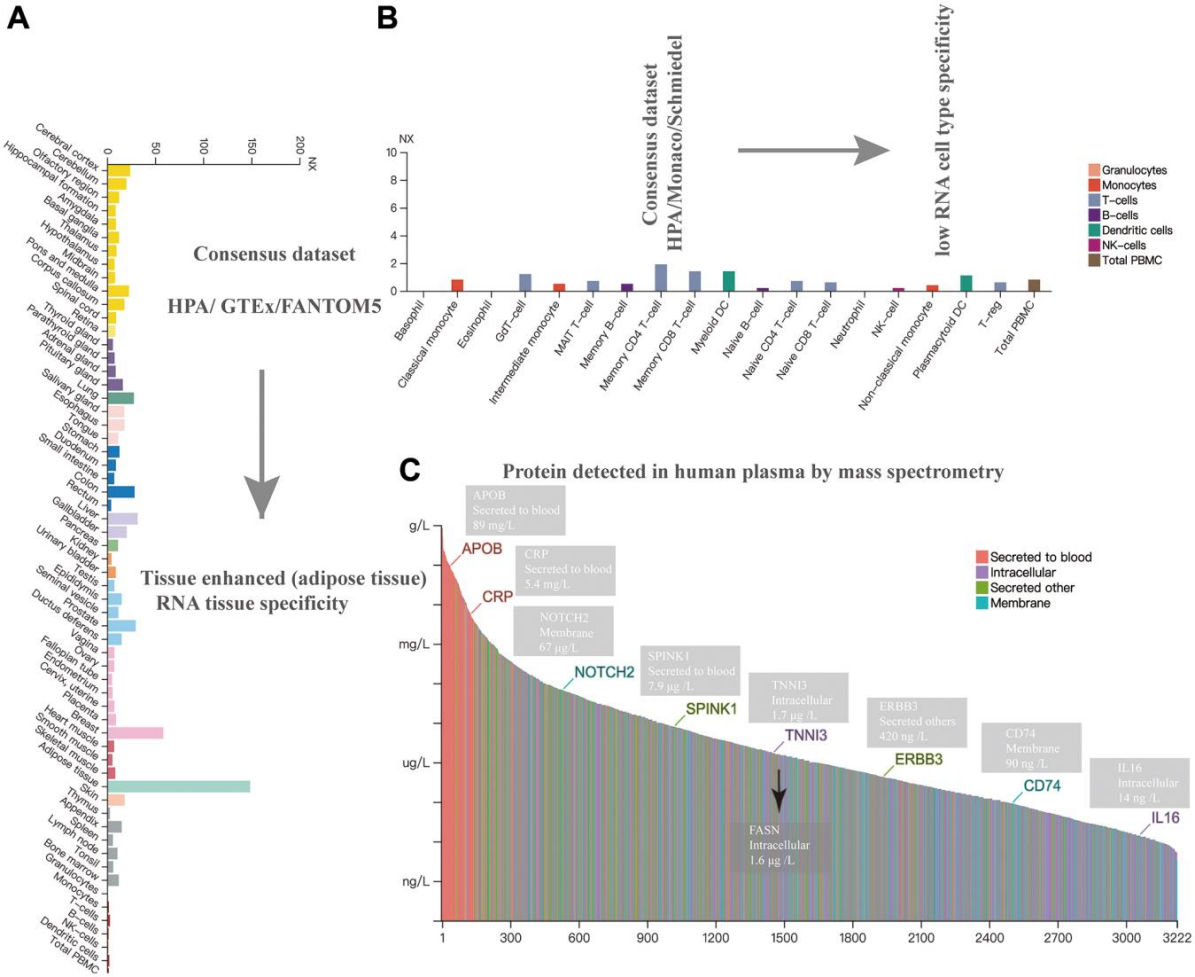
**Expression analysis of FASN in different cells, tissues, and plasma.** Expression of the *FASN* gene in different tissues (**A**), blood cells (**B**), and plasma (**C**) based on mass spectrometry.

The expression level of FASN in various cells/nontumor tissues/plasma was determined. FASN had the highest expression in the adipose tissue, followed by the breast and liver (Figure 2A). FASN was identified in all tissues, except granulocytes, and displayed tissue-enhanced (adipose tissue) RNA tissue specificity. Low RNA blood cell type specificity was demonstrated in various blood cells (Figure 2B). The protein density of FASN in the plasma was 1.6 μg/L, which might be due to the biological external leakage of the intracellular compound (Figure 2C).

**Figure 3:**
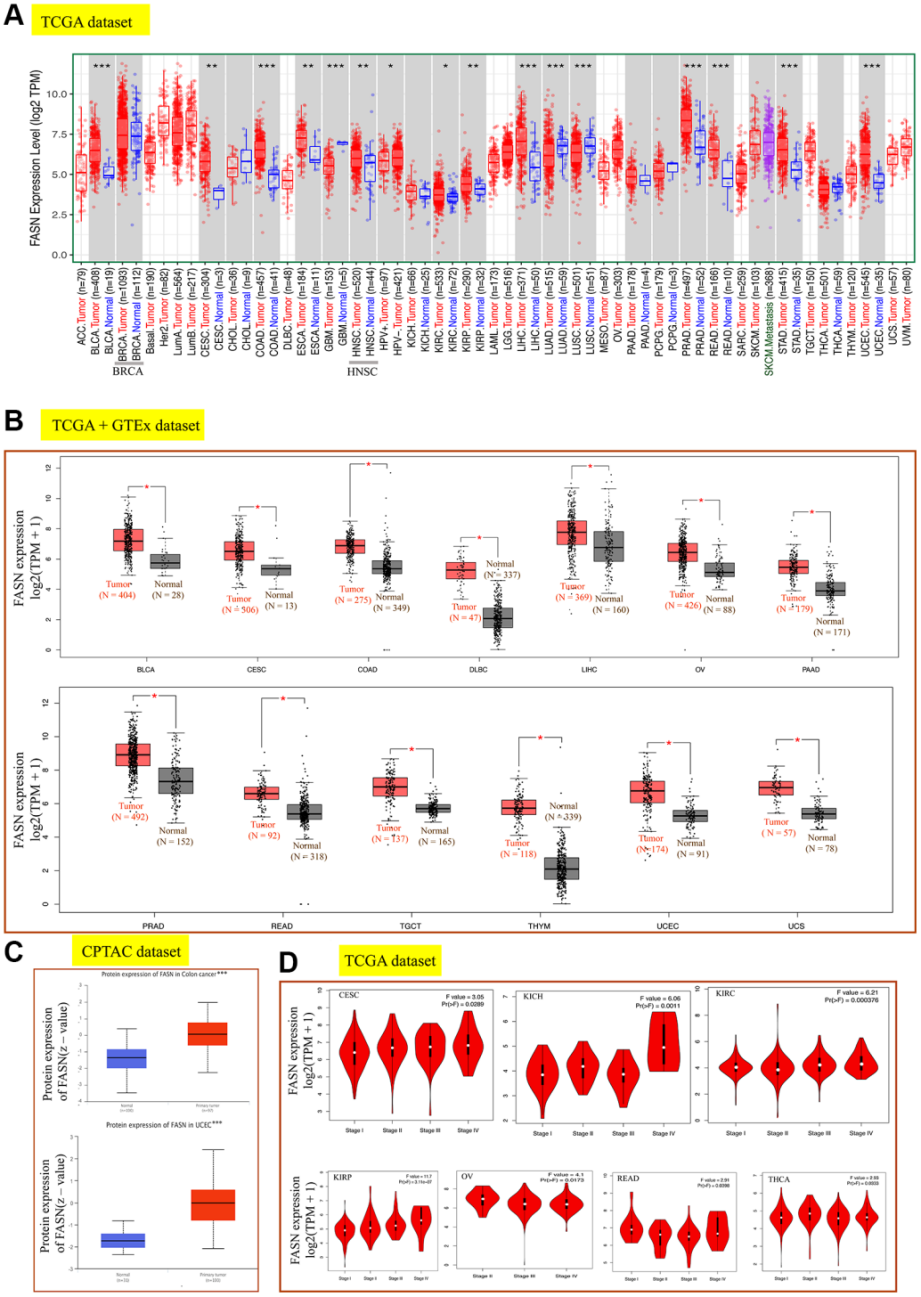
**Expression analysis of the *FASN* gene across various tumors and pathological stages.** (**A**) Expression analysis of the *FASN* gene in various cancers. (**B**) After employing the GTEx database, the box plot data were retrieved. (**C**) Expression analysis of FASN total protein between the normal group and the colon cancer and UCEC group. (**D**) Expression analysis of the *FASN* gene was performed according to the pathological stages. ^*^P < 0.05; ^**^P < 0.01; ^***^P < 0.001.

We analyzed the expression status of FASN in cancers. The expression level of FASN in the tissues of bladder urothelial carcinoma (BLCA), COAD, LIHC, PRAD, READ (Rectum adenocarcinoma), Stomach adenocarcinoma (STAD), Uterine Corpus Endometrial Carcinoma (UCEC) (*P* < 0.001), CESC, esophageal carcinoma (ESCA), head and neck squamous cell carcinoma (HNSC), kidney renal papillary cell carcinoma (KIRP) (*P* < 0.01), and kidney renal clear cell carcinoma (KIRC) (*P* < 0.05) was greater than that in comparable tissues (Figure 3A).

**Figure 4:**
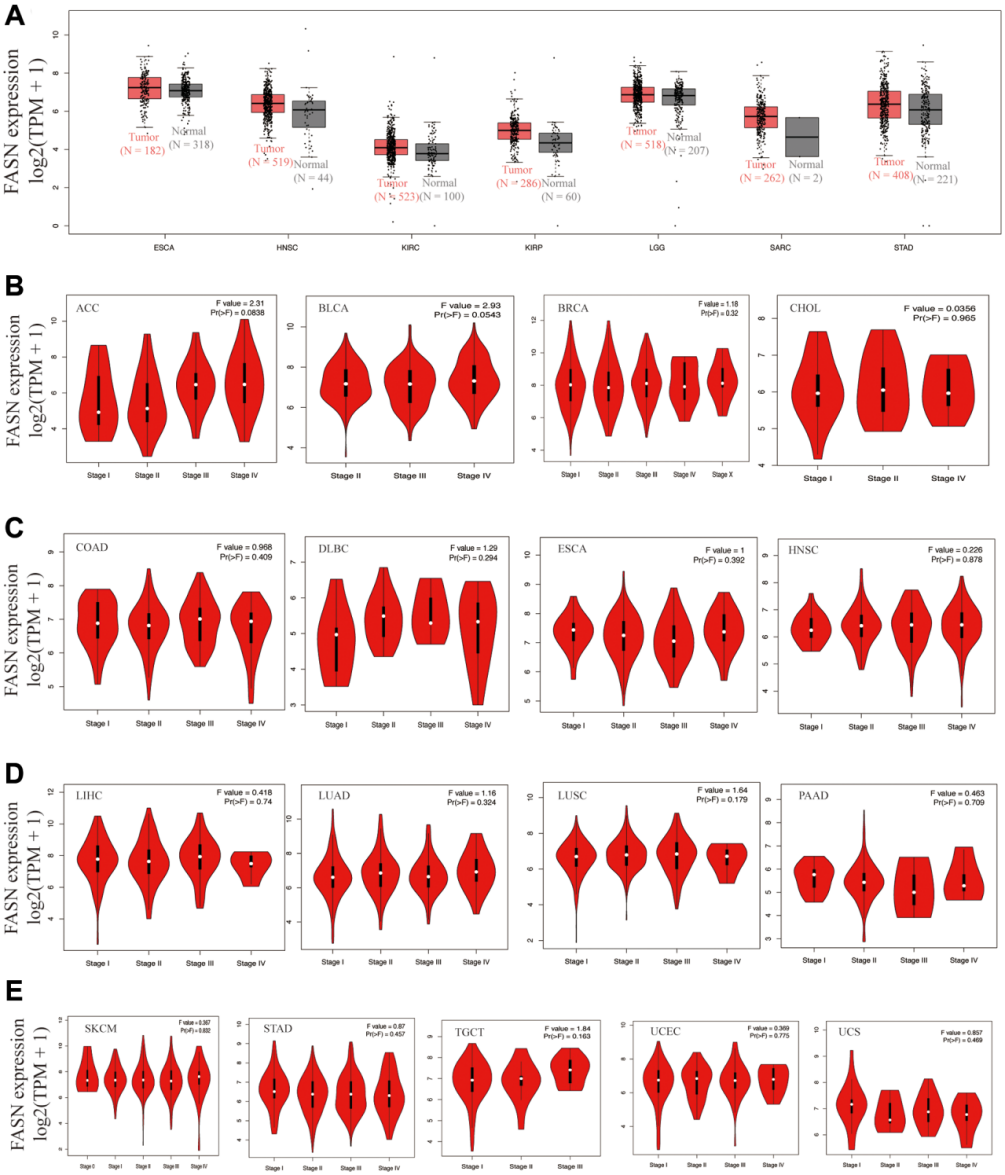
**Expression analysis of the *FASN* gene across various tumors and pathological stages.** (**A**) Expression analysis of the *FASN* gene in ESCA, HNSC, KIRC, KIRP, LGG, SARC, and STAD based on the GTEx databases. Expression analysis of the different pathological stages of ACC, BLCA, BRCA, CHOL (**B**); COAD, DLBC, ESCA, HNSC (**C**); LIHC, LUAD, LUSC, PAAD (**D**); and SKCM, STAD, TGCT, UCEC, UCS (**E**).

By incorporating the GTEx dataset, the expression level between the tumor-normal pair tissues was further identified in BLCA, CESC, COAD, lymphoid neoplasm diffuse large B-cell lymphoma (DLBC), LIHC, ovarian serous cystadenocarcinoma (OV), pancreatic adenocarcinoma (PAAD), PRAD, READ, testicular germ cell tumors (TGCT), thymoma (THYM), UCEC, and uterine carcinosarcoma (UCS) (Figure 3B, *P* < 0.05). However, a significant difference was not found for other tumors, such as ESCA, HNSC, LGG (brain lower grade glioma), or SARC (sarcoma) (Figure 4A).

**Figure 5:**
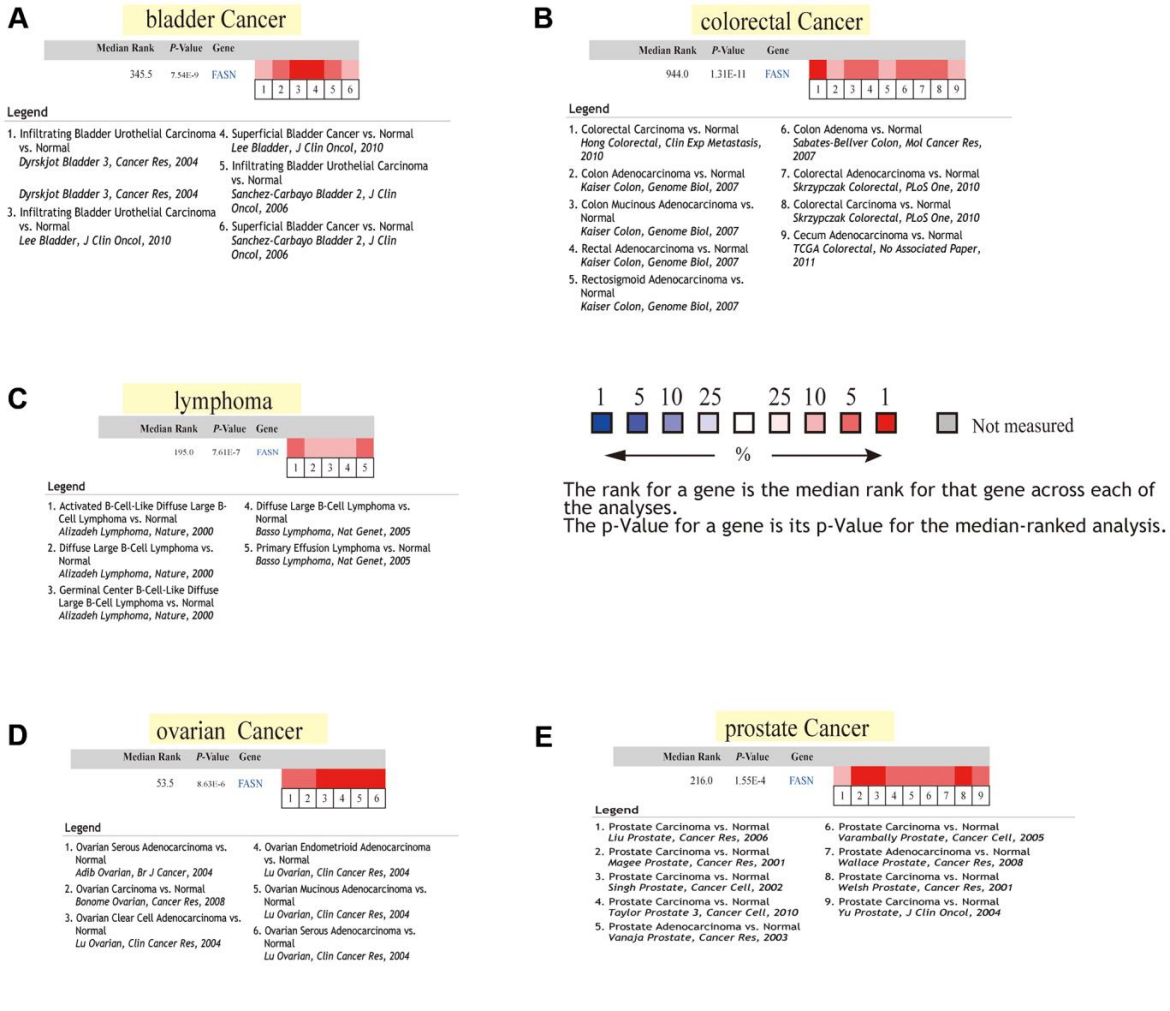
**Pooled FASN analysis using normal and tumor tissues.** (**A**) Lung cancer; (**B**) kidney cancer; (**C**) colorectal cancer; (**D**) lymphoma; and (**E**) myeloma.

Increased FASN expression was found in colon cancer and UCEC (Figure 3C, *P* < 0.001) relative to comparable tissues from the CPTAC dataset. The results of the pooling analysis (Figure 5) further affirmed that FASN expression was higher in bladder cancer, colorectal cancer, lymphoma, ovarian cancer, and prostate cancer tissues (*P* < 0.001) than in corresponding normal controls. A correlation was also found between FASN expression and the pathological stages of CESC, KICH, KIRC, KIRP, OV, READ, and thyroid carcinoma (THCA) (Figure 3D, *P* < 0.05); however, no correlation was found for other tumors (Figure 4B–4E).

### Immunohistochemistry analysis

**Figure 6:**
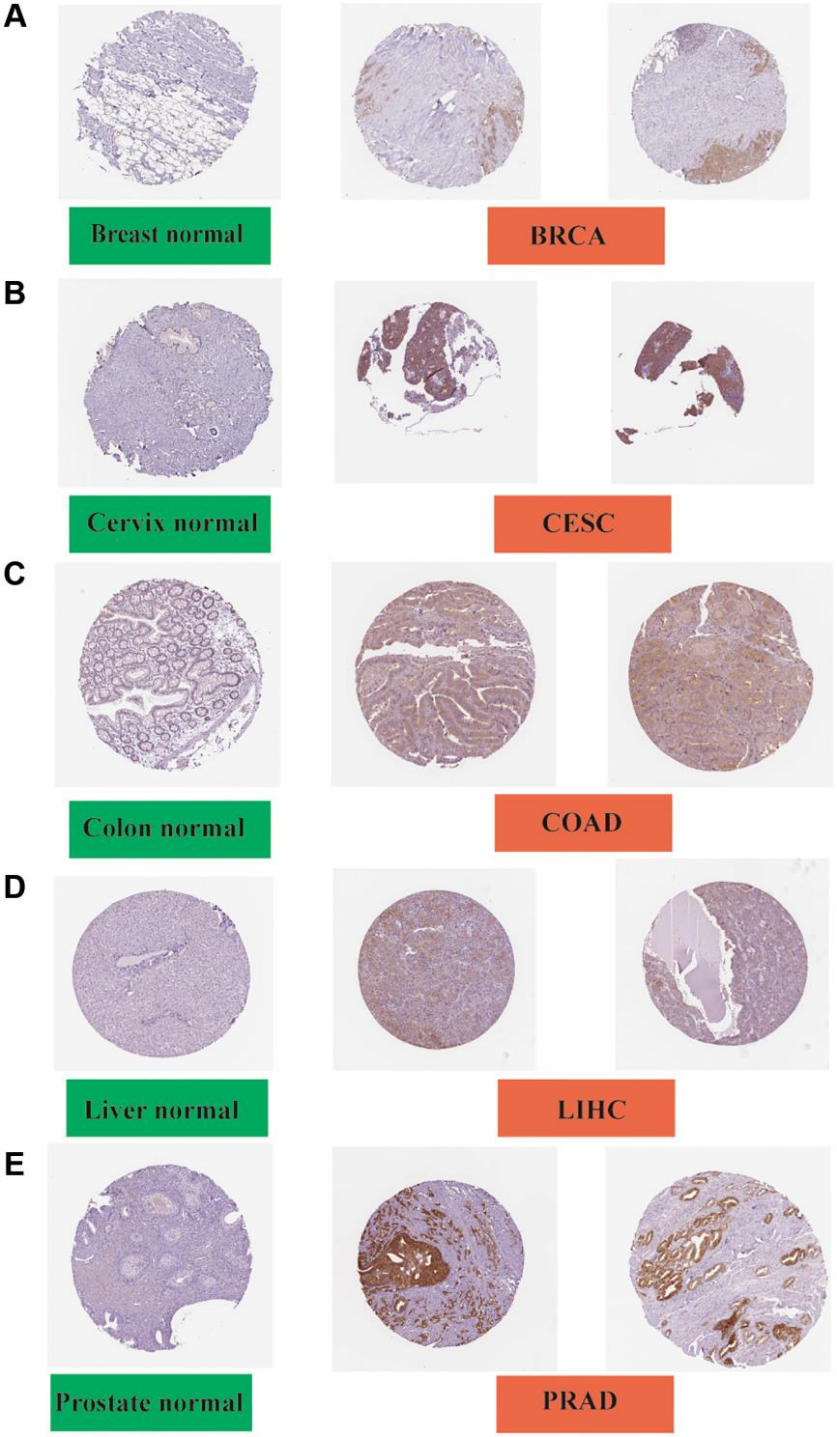
**Immunohistochemistry slides of normal (left) and tumor (middle and right) tissues.** FASN protein expression was significantly higher in BRCA, CESC, COAD LIHC, and PRAD. (**A**) Breast; (**B**) Cervix; (**C**) Colon; (**D**) Liver; and (**E**) Prostate.

The IHC results were obtained and the results of FASN gene expression in tumor tissues were compared with those in normal tissues. The gene expression data were found to be consistent with those of IHC. Negative or low IHC staining was obtained in the normal breast, cervix, colon, liver, and prostate tissue, while medium or strong staining was obtained in the cancer tissue (Figure 6).

### Survival analysis data

**Figure 7:**
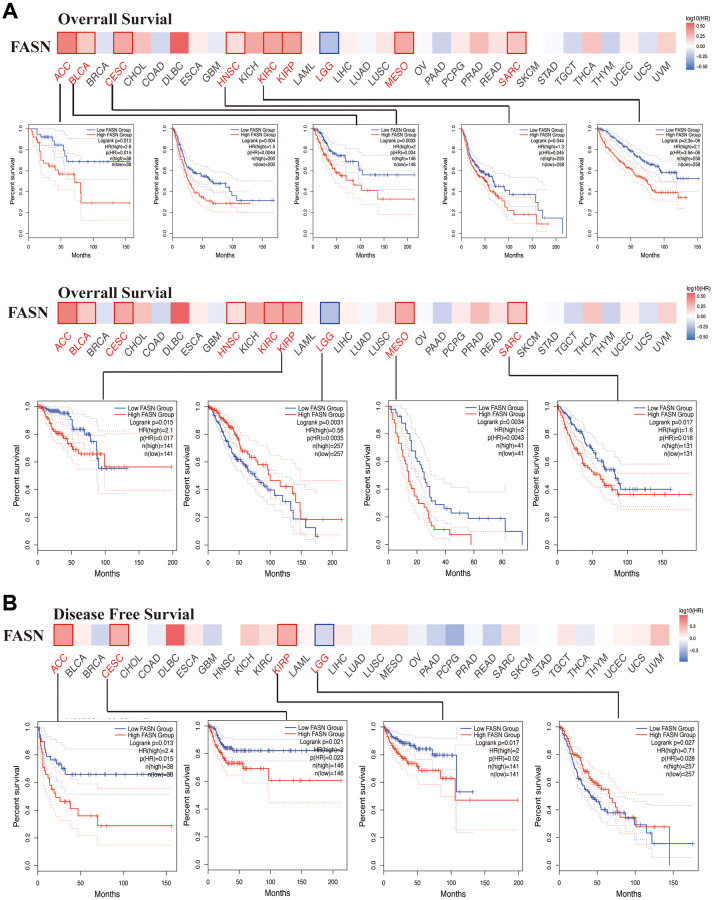
**Correlation between FASN gene and the survival outcome of tumors.** OS (**A**) and DFS (**B**) analyses of various tumors. The survival map and Kaplan-Meier curves with positive results are displayed.

We explored the relationship between FASN expression and the outcome of patients in the high and low expression groups. As shown in Figure 7A, high expression of FASN was related to poor OS outcomes in ACC (*p* = 0.015), BLCA (*p* = 0.0044), CESC (*p* = 0.004), HNSC (*p* = 0.045), KIRC (*p* = 3.9e-06), KIRP (*P* = 0.017), MESO (Mesothelioma) (*P* = 0.0043), LGG (*P* = 0.0035), and SARC (*P* = 0.018). DFS analysis (Figure 7B) further revealed a relationship between high FASN expression and poor outcome of ACC (*p* = 0.015), CESC (*p* = 0.023), LGG (*p* = 0.028), and KIRP (*p* = 0.02).

**Figure 8:**
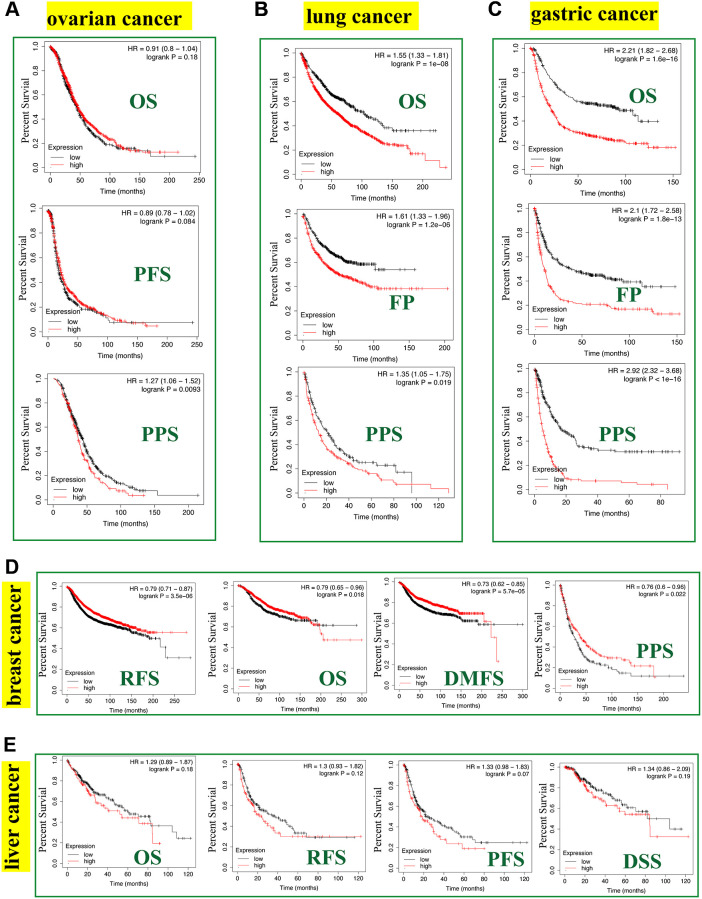
**Correlation between the *FASN* gene and the survival outcome of tumors.** We used the Kaplan-Meier plotter to carry out the survival analyses of *FASN* gene in breast cancer (**A**), ovarian cancer (**B**), lung cancer (**C**), gastric cancer (**D**), and liver cancer (**E**) cases.

**Figure 9:**
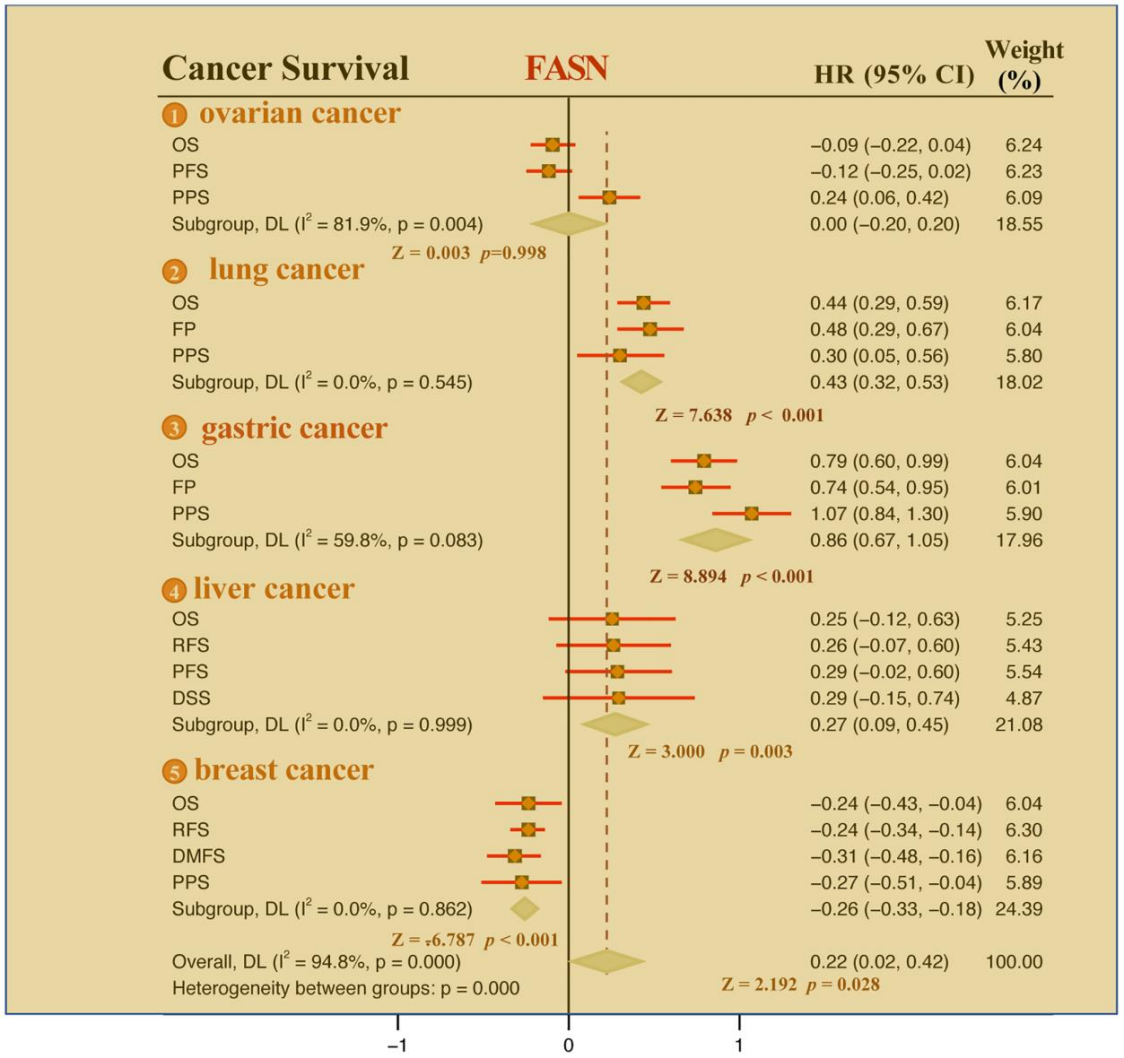
Meta-analysis of the correlation between FASN and the outcomes of breast cancer, ovarian cancer, lung cancer, gastric cancer, and liver cancer cases.

The Kaplan-Meier plotter tool revealed that FASN was associated with poor RFS (*P* = 0.0093) outcome for ovarian cancer (Figure 8A) and poor OS (*P* = 1e-08), FP (*P* = 1.2e-06), and PPS (*P* = 0.019) prognosis for lung cancer (Figure 8B). Additionally, a high FASN level was found to have a significant effect on the poor OS (*P* = 1.6e-16), FP (*P* = 1.8e-13), and PPS (*P* < 1e-16) outcomes for gastric cancer (Figure 8C). In contrast, a significant relationship between low expression of FASN and poor OS (*P* = 0.018), poor RFS (*P* = 3.5e-06), poor DMFS (*P* = 5.7e-05), and poor PPS (*P* = 0.022) outcomes was found for breast cancer (Figure 8D). In fact, a high expression of FASN was associated with poor OS, RFS, DMFS, and PPS (Supplementary Table 1, *P* < 0.05) for breast cancer patients with HER2 negative. Although we carried out further studies of liver cancer, a significant relationship between FASN and OS, PFS, RFS, and DSS outcomes was not found (Figure 8E, *P* < 0.05). Nonetheless, the results of the meta-analysis (Figure 9) confirmed the significant relationship between FASN expression and the outcomes for breast cancer, liver cancer, lung cancer, and gastric cancer (*P* < 0.05) but not for ovarian cancer (*P* = 0.998). Subgroup analyses were also performed with the clinical characteristics and definite results were obtained (Supplementary Tables 1–5). Overall, the findings revealed that FASN is dissimilarly associated with the outcome of cancer cases.

### Genetic alteration analysis

**Figure 10:**
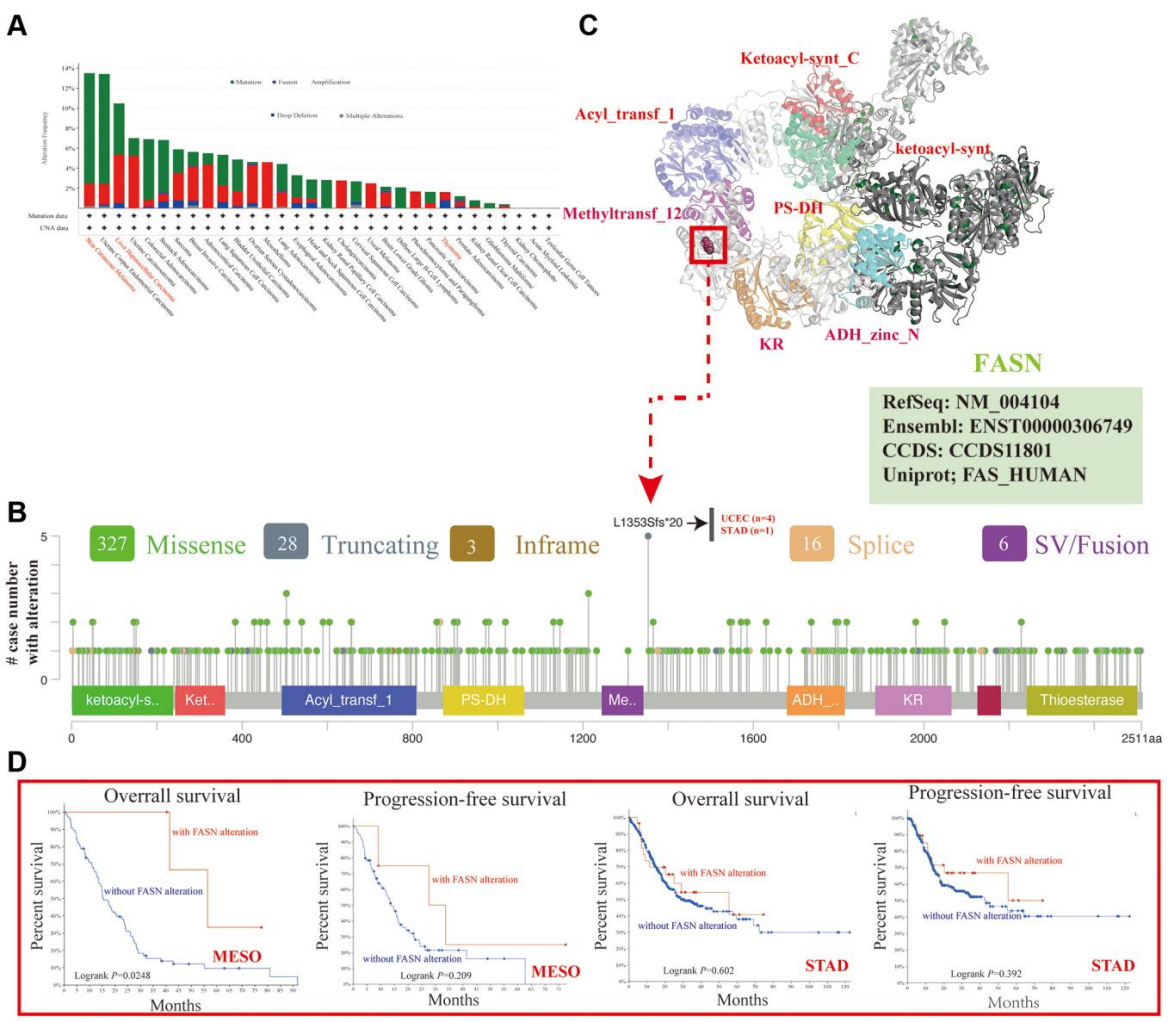
**Mutation feature of FASN across various tumors.** The alteration frequency with mutation type (**A**) and mutation site (**B**) is displayed. The mutation site with the highest alteration frequency (L1353Sfs*20) in the 3D structure of FASN (**C**). The potential correlation between mutation status and the OS and PFS of MESO and STAD (**D**).

The genetic alteration level of FASN has been observed in various cancers. The highest alteration frequency of FASN (>11%) was found in patients with Skin Cutaneous Melanoma with “mutation” (Figure 10A). The CNA data of liver HCC showed a >5% alteration incidence (Figure 10A). However, the value in thymoma cases (~1% genetic alteration frequency) was not detected (Figure 10A). Figure 10B shows the types, sites, and number of FASN genetic alterations. The missense mutation of FASN is the principal type of genetic alteration. L1353Sfs*20 alteration (four cases of uterine endometrioid carcinoma and one case of stomach adenocarcinoma) between Methyltransf_12 and ADH_zinc_N could induce a translation mutation from L (leucine) to S (serine) at the 1353 site of the FASN protein (Figure 10B). The L1353 locus was identified in the 3D protein structure (Figure 10C). Moreover, the relationship between genetic alterations and clinical outcomes of cancers was investigated.

Figure 10D shows that MESO patients with altered FASN had better outcomes for OS (*P* = 0.0248), but not for PFS (*P* = 0.209) while STAD patients had better OS (*P* = 0.602) and PFS (*P* = 0.392) than patients without FASN alteration.

### TMB/MSI analysis

**Figure 11:**
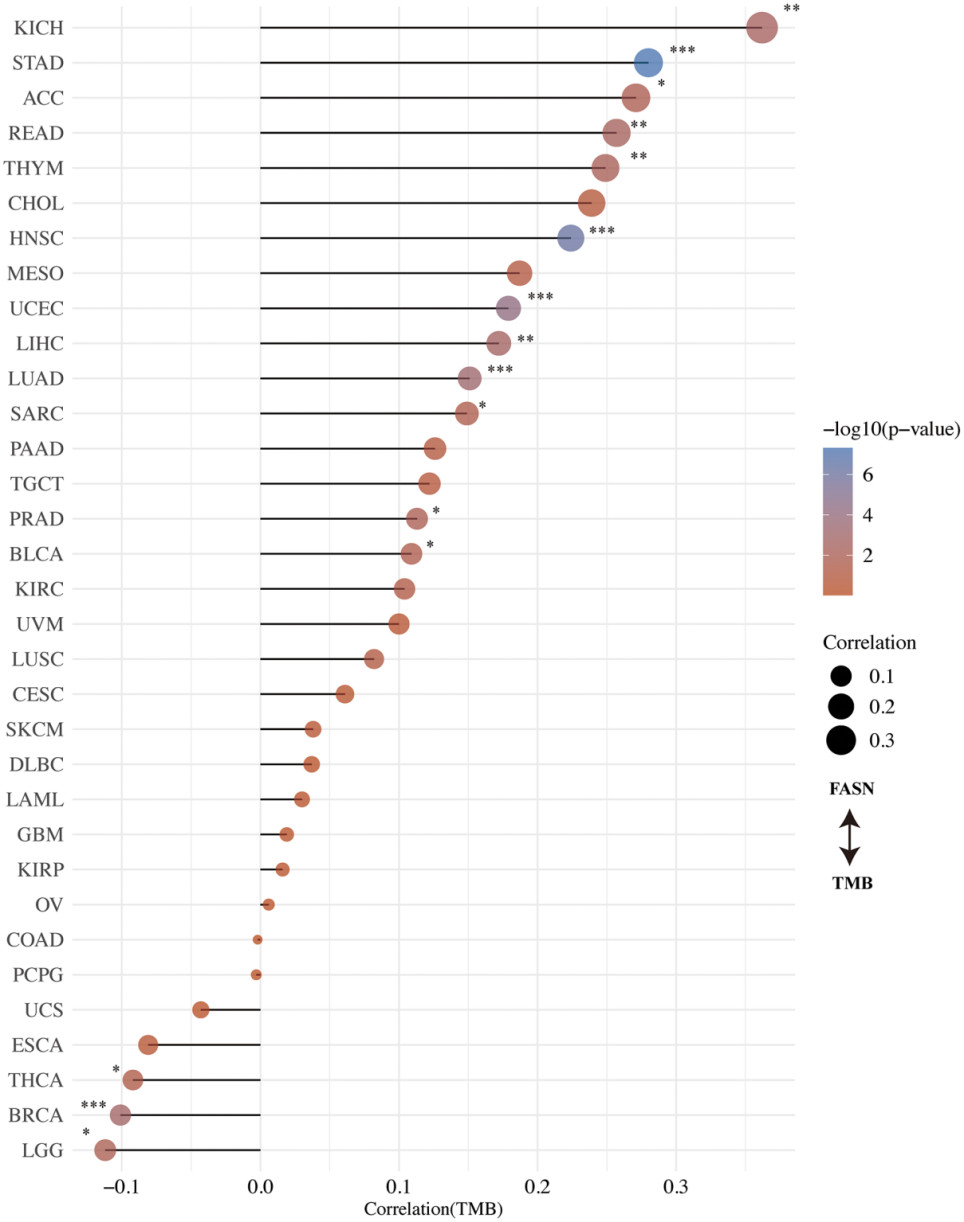
Correlation between FASN expression and tumor mutational burden.

**Figure 12:**
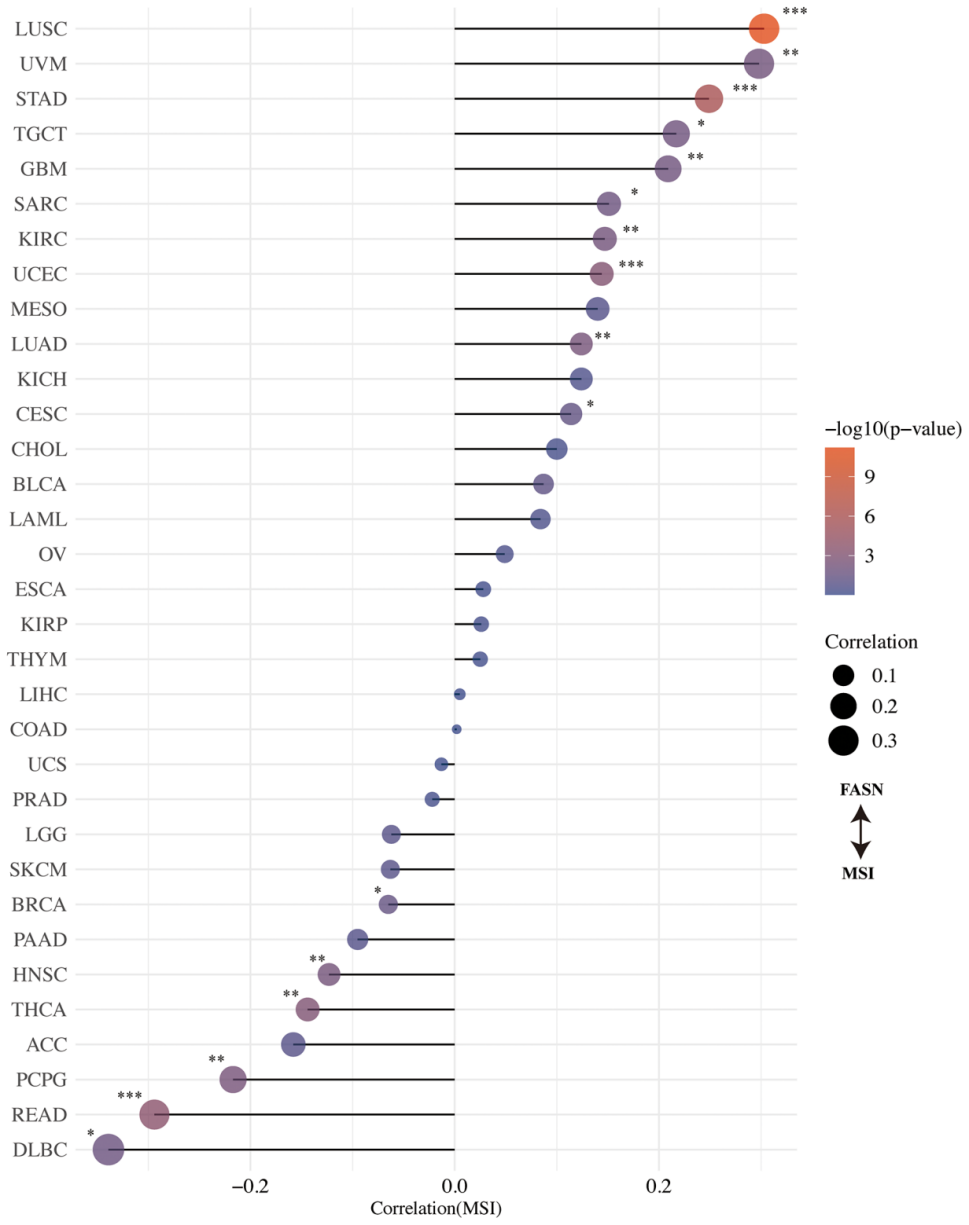
Correlation between FASN expression and microsatellite instability.

The correlation between FASN expression and TMB/MSI was determined. An adverse significant association was found between FASN and TMB for STAD, HNSC, UCEC, LUAD, LIHC, KICH, READ, THYM, PRAD, ACC, SARC, and BLCA, while a significant association was found for BRCA, LGG, and THCA (Figure 11). With respect to MSI, DLBC, READ, PCPG, THCA, HNSC, and BRCA were found to have a significant negative correlation, while CESC, LUAD, UCEC, KIRC, SARC (Sarcoma), GBM, TGCT, STAD, UVM, and LUSC had a significant positive correlation (Figure 12). More studies are required to explain this phenomenon.

### DNA methylation analysis

**Figure 13:**
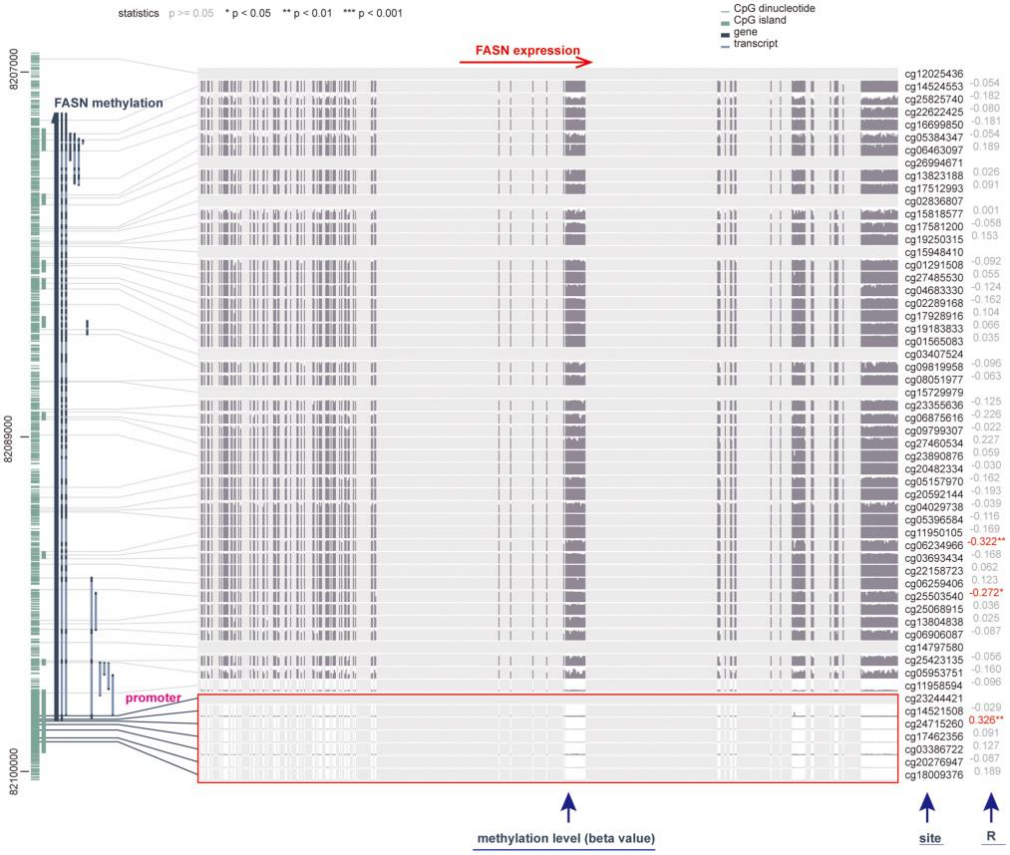
**Correlation between DNA methylation and FASN expression in GBM cases.** The promoter region is selected.

**Figure 14:**
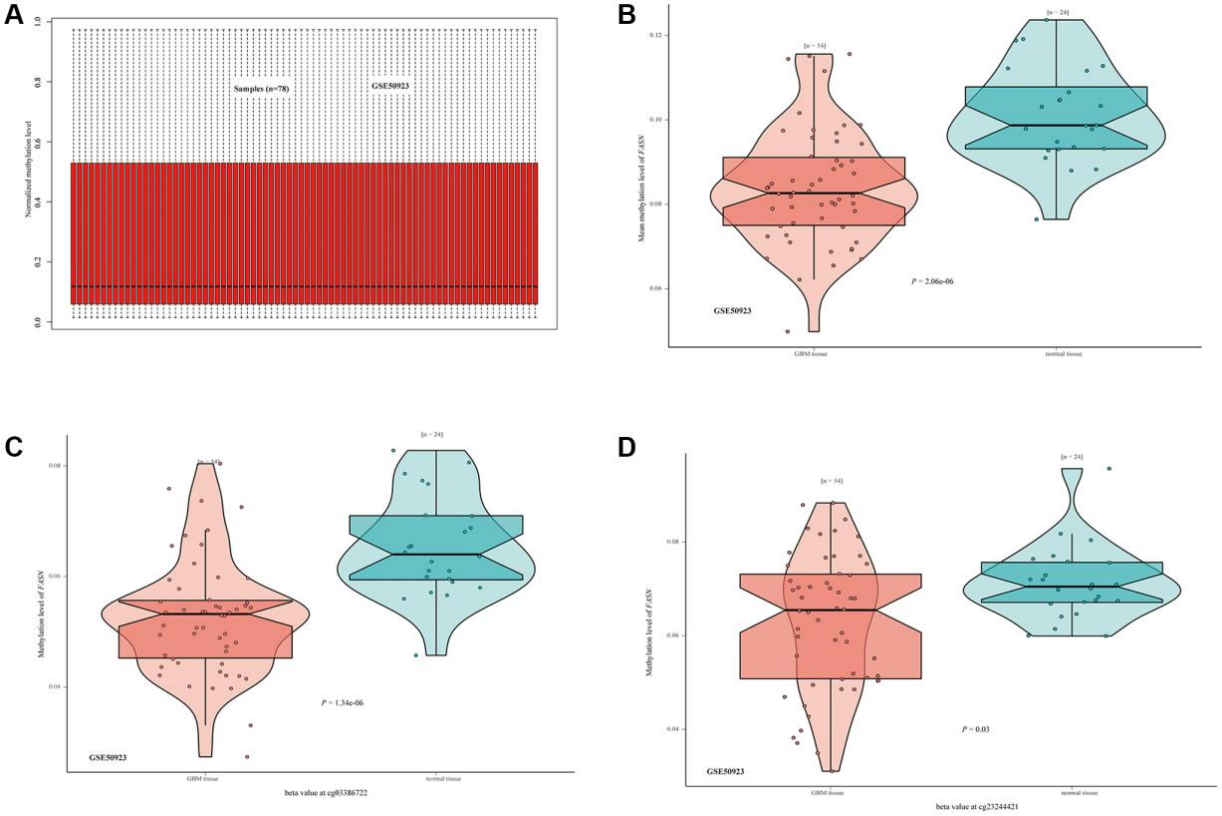
**DNA methylation status of FASN in the GSE50923 dataset.** (**A**) Normalization of the GSE50923 dataset; (**B**) methylation status of FASN in the GBM group and the matched normal group; (**C**, **D**) beta value of FASN in cg03386722 and cg23244421.

We investigated the correlation between FASN DNA methylation and tumorigenesis in various cancers. With respect to GBM, we obtained a significant adverse relationship between DNA methylation and gene expression at probes of the non-promoter region, such as cg06234966 (*P* < 0.01, R = 0.322) (Figure 13). The GSE50923 dataset was used to confirm these results. In light of the standard methylation result (Figure 14A), we neglected the difference between GBM tissues and normal tissues (Figure 14B, *P* = 2.06e-06). Furthermore, we observed a decrease in the methylation level of FASN in cancer tissue compared to normal tissue for the selected probes (Figure 14C, cg03386722, *P* = 1.34e-06, Figure 14D, cg23244421, *P* = 0.03).

### Protein phosphorylation analysis

**Figure 15:**
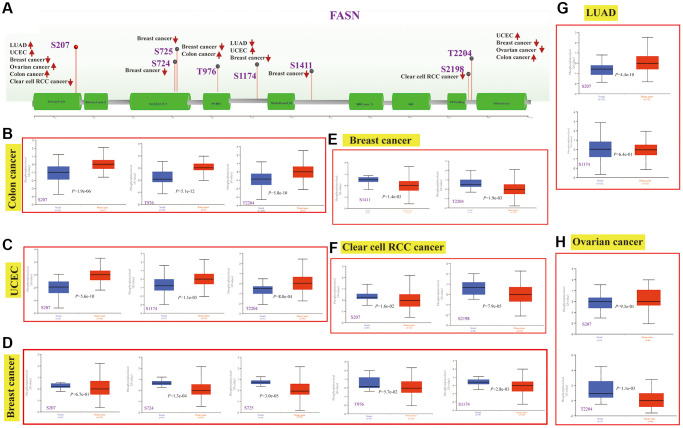
**Phosphorylation analysis of the FASN protein.** Phosphoprotein sites of the FASN protein are indicated in the schematic (**A**). Expression analysis of FASN phosphoprotein (NP_004595.4, S207, S724, S725, T976, S1174, S1411, S2198 and T2204 sites) between normal tissue and tumors, including colon cancer (**B**), UCEC (**C**), breast cancer (**D**, **E**), clear cell RCC (**F**), LUAD (**G**), and ovarian cancer (**H**).

FASN phosphorylation levels between normal tissues and the corresponding tumor group were obtained, and six types of cancers (breast cancer, clear cell RCC, LUAD, ovarian cancer, colon cancer, and UCEC) were analyzed. Figure 15A summarizes the FASN phosphorylation sites among these cancers.

The S207 sites within the ketoacyl-synt domain of FASN showed significant difference phosphorylation levels in tumor tissue compared to normal tissue (Figure 15B–15D, Figure 15F–15H, all *P* < 0.05), followed by the T2204 sites between PP-binding and thioesterase domain for colon cancer (Figure 15B, *P* = 5.8e-10), UCEC (Figure 15C, *P* = 8.0e-04) and breast cancer (Figure 15E, *P* = 1.9e-03). The PhosphoNET database revealed that FASN phosphorylation of S207 in the molecular responses to pathway activation was experimentally supported by previous investigations [20, 21] (Supplementary Table 6). Such finding aided in the exploration of the cancer role of S207 phosphorylation in tumors.

### Immune infiltration analysis

**Figure 16:**
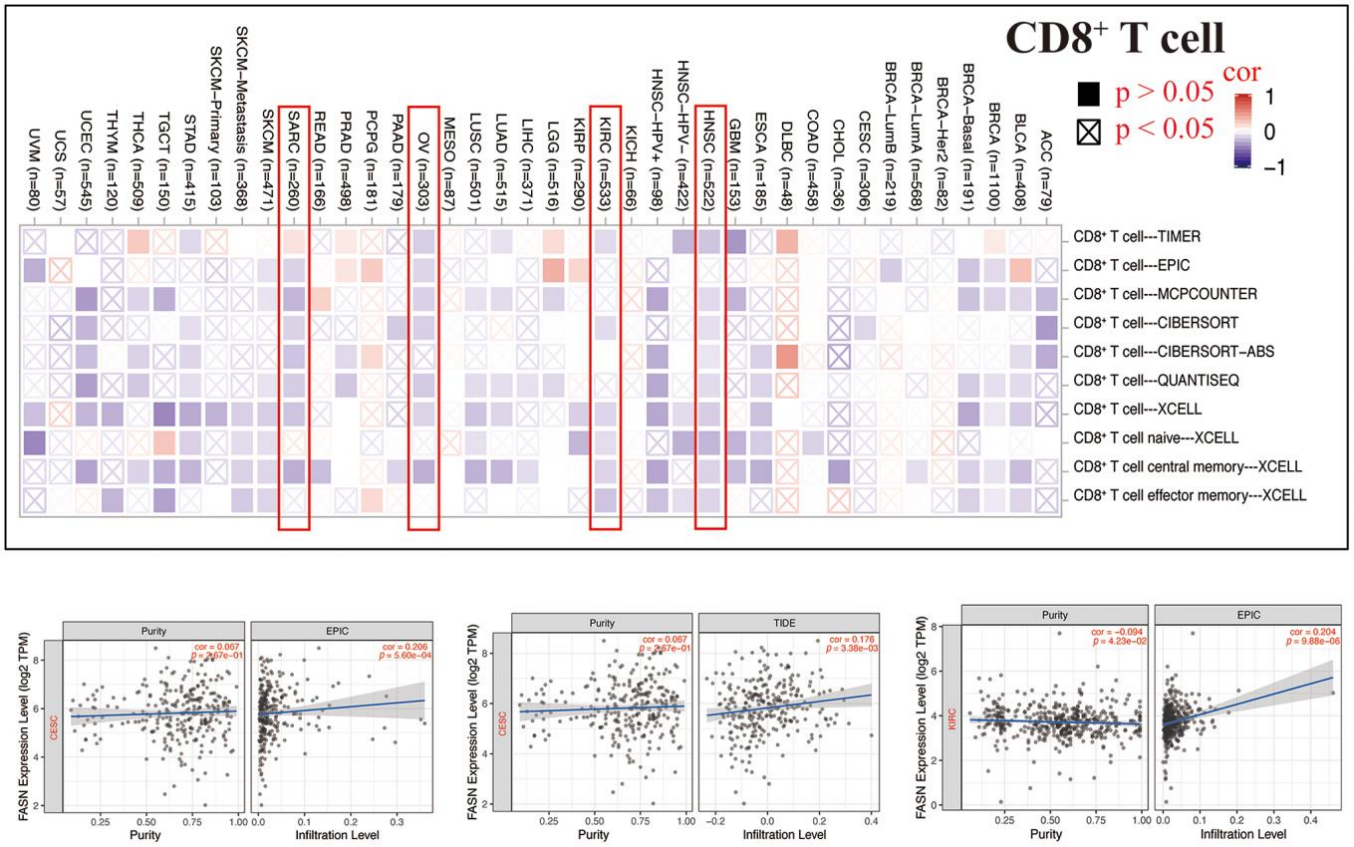
Relationship between FASN expression and CD8+ T-cells.

**Figure 17:**
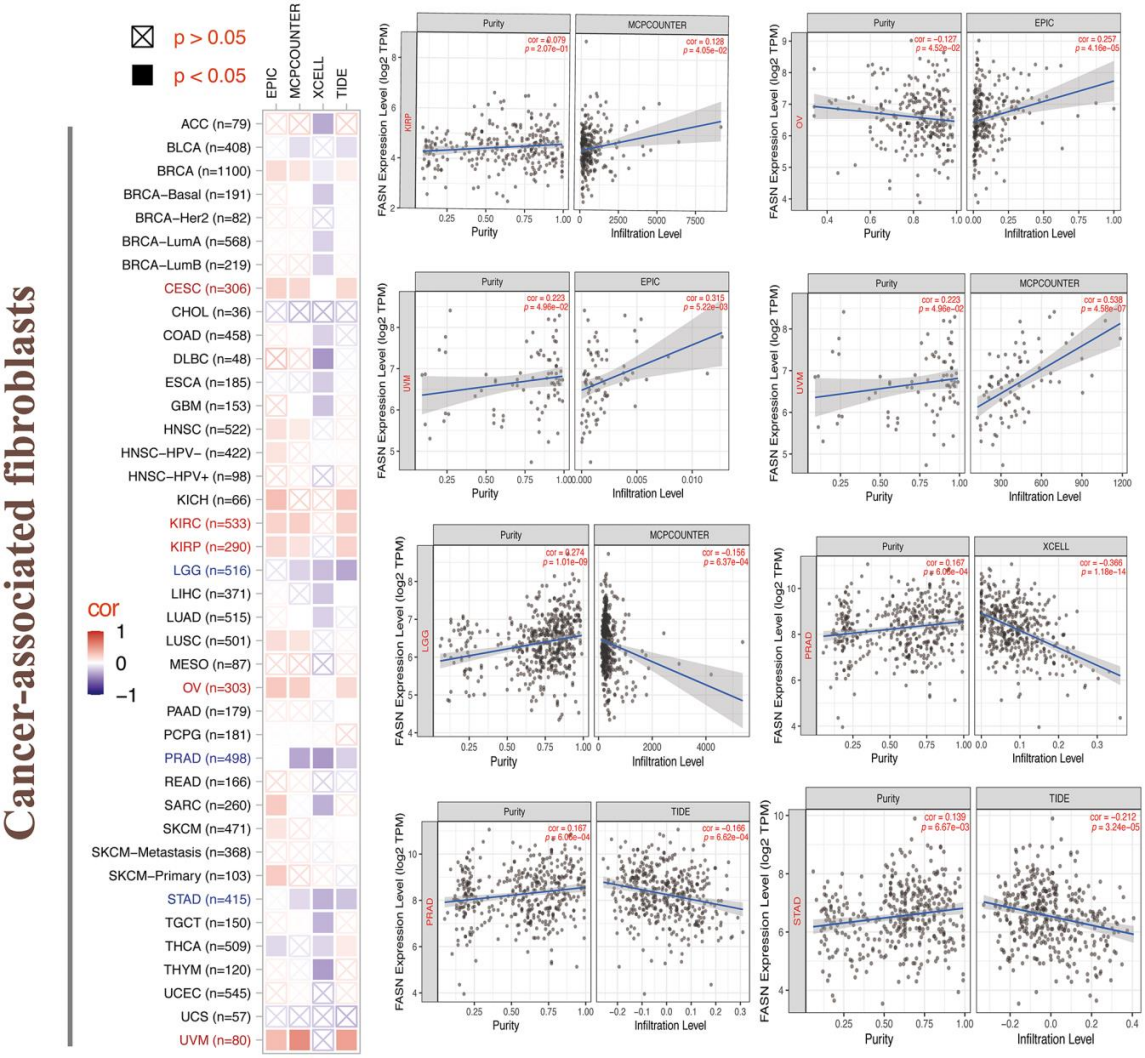
Relationship between FASN expression and cancer-associated fibroblasts.

Tumor-infiltrating immune cells can enhance the development, progression, or metastasis of cancers [22, 23]. A statistically significant adverse relationship was found between CD8^+^ T cells and FASN expression in HNSC, KIRC, OV, and SARC (Figure 16), depending on all/most algorithms. Further, a statistically significant relationship was presumed between FASN expression and cancer-associated fibroblasts for CESC, KIRC, KIRP, OV, and UVM; however, a negative correlation was presumed for LGG, PRAD, and STAD (Figure 17). A negative correlation was also found between FASN expression and CD4+ T cells and NK cells in BLCA, BRCA, and THCA, while a positive correlation was found in HNSC (Supplementary Table 7).

### Enrichment analysis of FASN-related partners

**Figure 18:**
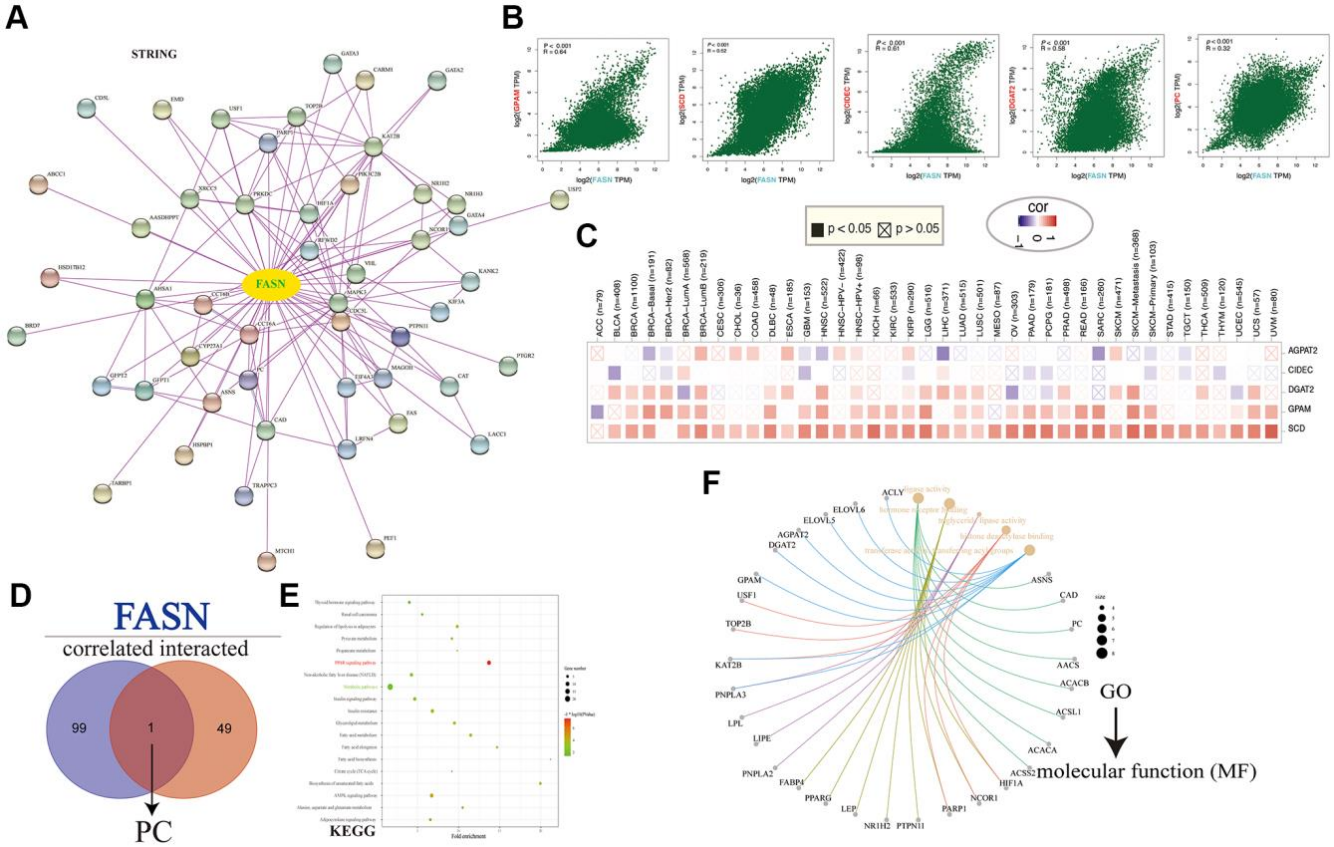
**FASN-related gene enrichment analysis.** (**A**) FASN-binding proteins. (**B**) Expression correlation between FASN and GPAM, SCD, CIDEC, DGAT2, and PC. (**C**) Corresponding heatmap across cancers. (**D**) The intersection of the FASN-binding and correlated genes. (**E**) KEGG pathway analysis. (**F**) GO (Molecular function) analysis.

We screened out FASN-binding proteins and FASN-expression-correlated genes for functional enrichment analyses. We collected 51 FASN-binding proteins using the STRING tool (Figure 18A). FASN expression was positively associated with five genes. The top 100 FASN-correlated targeting genes included glycerol-3-phosphate acyltransferase, mitochondrial (*GPAM*) (R = 0.64), stearoyl-CoA desaturase (SCD) (R = 0.62), cell death inducing DFFA-like effector c (CIDEC) (R = 0.61), diacylglycerol O-acyltransferase 2 (DGAT2) (R = 0.58), and pyruvate carboxylase (PC) (R = 0.32) (*P* < 0.001) (Figure 18B). The heatmap revealed a positive correlation between FASN and the five genes across cancer types (Figure 18C). PC was the only intersection member of the two groups (Figure 18D).

**Figure 19:**
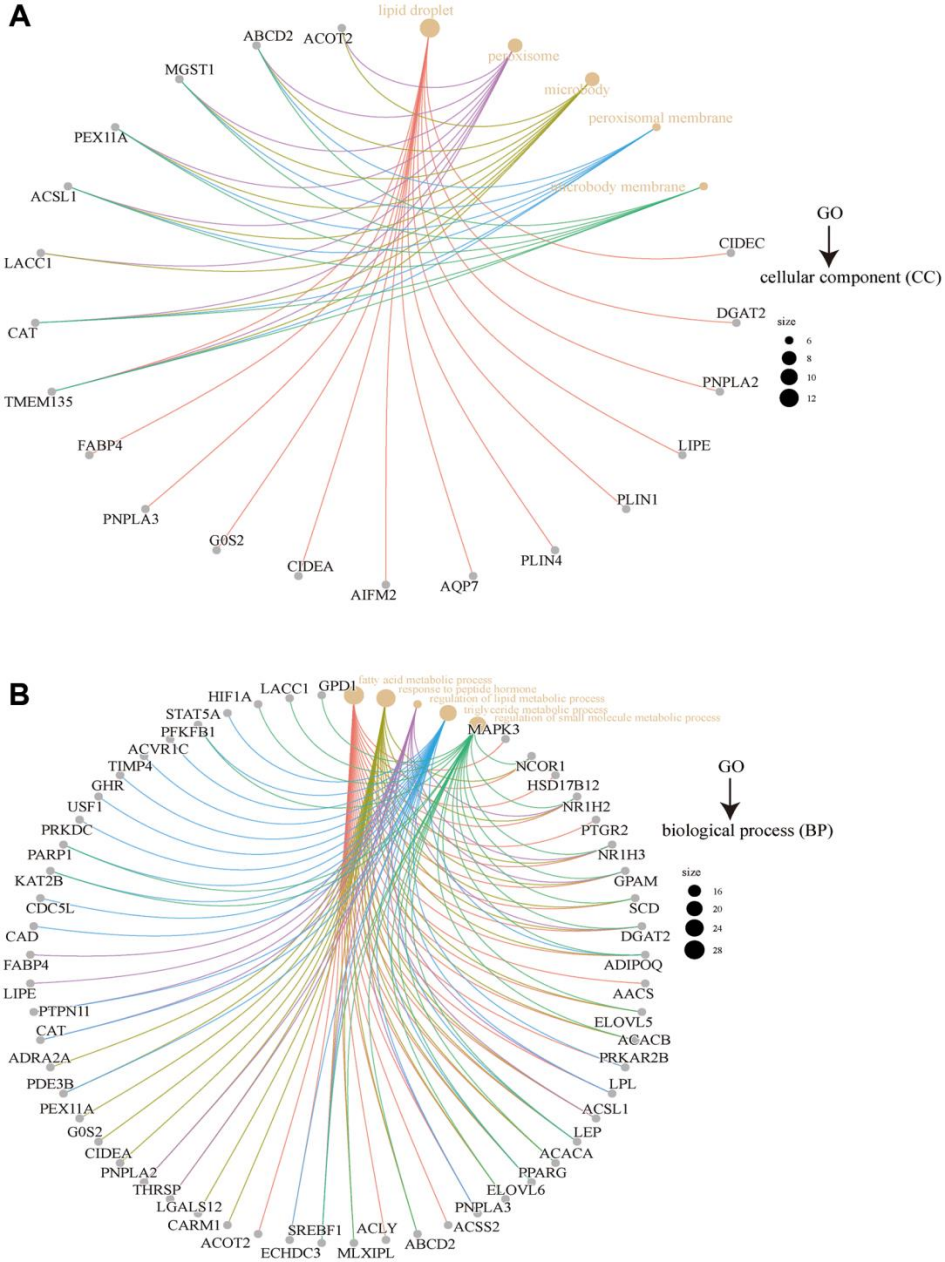
**GO analysis of FASN-related genes.** Results for cellular component (**A**) and biological process (**B**).

The KEGG analysis resulted presented in Figure 18E revealed that “metabolic pathways” and “PPAR signaling pathway” might play a role in the effect of FASN on tumorigenesis. GO analysis further suggested that these genes were mainly enriched in lipid metabolism, such as triglyceride lipase activity, lipid droplets, fatty acid metabolic processes, regulation of lipid metabolic processes, and others (Figures 18F and 19).

## DISCUSSION

The FASN protein participates in multiple functions in biological processes across various species, such as gene axis, maturation of Treg cells, and cell apoptosis [24–26]. Several studies have revealed a functional connection between FASN and cancers. However, whether FASN can play a role in the tumorigenesis of cancers through these mechanisms remains unclear. Initially, the “HomoloGene” and phylogenetic tree suggested the conservation of the protein structure of FASN among various species, indicating that similar mechanisms may exist as a regular physiological role.

FASN expression was found to be higher in most tumor tissues than contrast tissues. However, survival outcome analysis yielded distinct conclusions for different tumors. Based on our analysis, FASN had a higher expression at the protein level in breast cancer. Based on prior studies, a high expression of FASN can increase the recurrence or metastasis rate of breast cancer [27–29]. Herein, high presentation was not found to be related to poor OS and DMFS outcomes in patients with breast cancer. However, high FASN expression was related to poor OS, RFS, DMFS, and PPS in breast cancer cases with HER2 negative subtype/mesenchymal (Supplementary Table 1). A recent study revealed that FASN has a higher expression in the HER2 positive subgroup than the HER2 negative group, and its inhibitor could prevent the agonistic tumor-promoting activity of tamoxifen and restore its estrogen antagonist properties against ER/HER2- positive xenograft tumors in mice [30]. Therefore, further clinical characteristics should be considered.

High FASN expression was not found to be related to poor OS in the TCGA-LUSC and TCGA-LUAD cohorts for lung cancer. To avoid incorrect conclusions resulting from FASN expression/clinical information processing, the OncoLnc database was used to perform a Cox regression survival analysis. FASN expression was found to be associated with lung squamous cell carcinoma (Cox coefficient = 0.182, *P* = 0.0085), but not lung adenocarcinoma. Similar with the results, high FASN expression was associated with poor OS, FP, and PPS in lung adenocarcinoma cases (Supplementary Table 2). Several studies have revealed that FASN can be activated to enhance ERK2 or promote lipogenesis associated with lung adenocarcinoma and it is also expressed in the bronchial epithelium and epithelial hyperplasia in lung squamous cell cancer [31, 32]. Consequently, larger sample sizes or clinical features may be required to confirm the role of FASN in different types of lung cancer.

The analysis of ovarian cancer suggests that a particular regulatory pathway may exist. Although FASN is important for the modulation of cell death in ovarian cancer cells by engaging in a caspase-2 regulatory mechanism [33], high FASN expression was not found to predict poor OS and DFS in ovarian serous cystadenocarcinoma. However, based on the GEO datasets of ovarian cancer, high expression of FASN was found to be related to poor OS in the stage/ stage 2/3/4 subgroup, PFS in the histology/serous or TP53/mutated subgroups, and PPS in the histology/serous, debulk/optimal, and chemotherapy/ containing platin subgroups (Supplementary Table 3). Consistently, although SIRT3 could enhance invasion and metastasis by improving the expression of FASN in cervical cancer [34], TCGA-based results did not reveal a correlation between high expression of FASN and poor outcome for UCEC.

FASN expression was found to be higher in the STAD group than in the control group. The high expression of FASN has an adverse effect on OS, FP, and PPS in gastric cancer. Accordingly, our results may provide a gene biomarker that could predict OS in patients with gastric cancer. Zhou et al. also reported that FASN is a prognostic marker related to immune infiltration in gastric cancers [35]. Herein, FASN was revealed to have a higher expression in LIHC tissues than the corresponding tissues. As hepatocellular carcinoma progression is highly dependent on FASN and its mediated lipogenesis [36], a relationship was found between FASN expression and OS, PFS, RFS, and DSS in liver cancer patients. A meta-analysis further affirmed that FASN may play a cancerous role in various tumors.

In this study, we determined the potential relationship between FASN expression and MSI/TMB across tumors. Prior studies have suggested a negative relationship between FASN expression and TMB or MSI and the clinical outcomes of READ and HNSC [37, 38]. For GBM patients, FASN was found to be highly expressed when the DNA methylation level in the non-promoter region was reduced. Similar findings were obtained for various methylation probes between the GBM group and the normal group of GSE50923. However, the different probes may produce different results. Moreover, glioma patients with isocitrate dehydrogenase mutations or wild-type patients had distinct molecular mechanisms (e.g., DNA repair pathways or anti-oxidative pathways) and distinct outcomes [39]. In conclusion, although FASN could enhance the malignant progression of GBM [40], more experiments and clinical trials are needed to verify the results.

The CPTAC dataset was employed to explore the mechanisms of protein and phosphoprotein change in the FASN protein in six types of tumors. Based on the findings, the S207 locus is highly expressed in tumors compared with normal tissues. S207 phosphorylation of FASN has been reported to be essential for molecular responses to pathway activation during biological processes [21, 41]. In addition, lysyl-tRNA synthetase is highly expressed in specific cellular compartments upon phosphorylation in the nucleus after S207 phosphorylation, leading to alternative noncanonical functions in colon cancer [42]. Previously, Motzik et al. found that Ap4A increased the oncogenic activity of melanoma patients and predict a poor outcome, and Ap4A was mainly produced by S207-phosphorylated lysyl-tRNA synthetase [43]. Nevertheless, the specific role of the phosphorylation level of the S207 locus in was not found. More laboratory studies are thus needed to further assess the oncogenic role of FASN phosphorylation and its related regulatory mechanism.

A statistically significant difference was found between FASN and CD8^+^ T-cells in HNSC, KIRC, OV, and BRCA (Figure 16). These results were consistent with those of published studies, which revealed that phosphatidylinositol 3-kinase α inhibitor could promote fatty acid metabolism to activate CD8+T cells, and a combination of the inhibitor and FASN inhibitor could enhance immunity to decrease breast tumor growth [44]. A favorable relationship was also found between FASN expression and cancer-associated fibroblasts in CESC, KIRC, KIRP, OV, and UVM. Furthermore, a negative correlation was found between FASN expression and CD4+ T cells and NK cells in BLCA, BRCA, and THCA, while a positive correlation was found in HNSC. In conclusion, we hypothesized that FASN plays an oncogenic role through an immune regulatory mechanism.

Based on enrichment analyses carried out using the integrated unit of FASN-binding components and expression-related genes, “hormone receptor binding,” “metabolic pathways,” and “fatty acid metabolic process” may play an important role in the progression of tumors. However, more experiments are needed to assess the oncogenic role of FASN.

Based on a complete analysis across tumors, we found a factual association between FASN expression and clinical outcome, DNA methylation, protein phosphorylation, immune cell infiltration, TMB, or MSI, which could help to better understand the oncogenic role of FASN.

## Supplementary Materials

Supplementary Figures

Supplementary Tables 1-6

Supplementary Table 7
