# Supplementary material for: Landscape of the oncogenic role of fatty acid synthase in human tumors
Source: Aging (Albany NY). 2021 Dec 8;13(23):25106–37. doi: 10.18632/aging.203730 (PMC8714155; doi:10.18632/aging.203730)
Supplement: Supplementary Figures [file aging-13-203730-s001.pdf]

SUPPLEMENTARY FIGURES

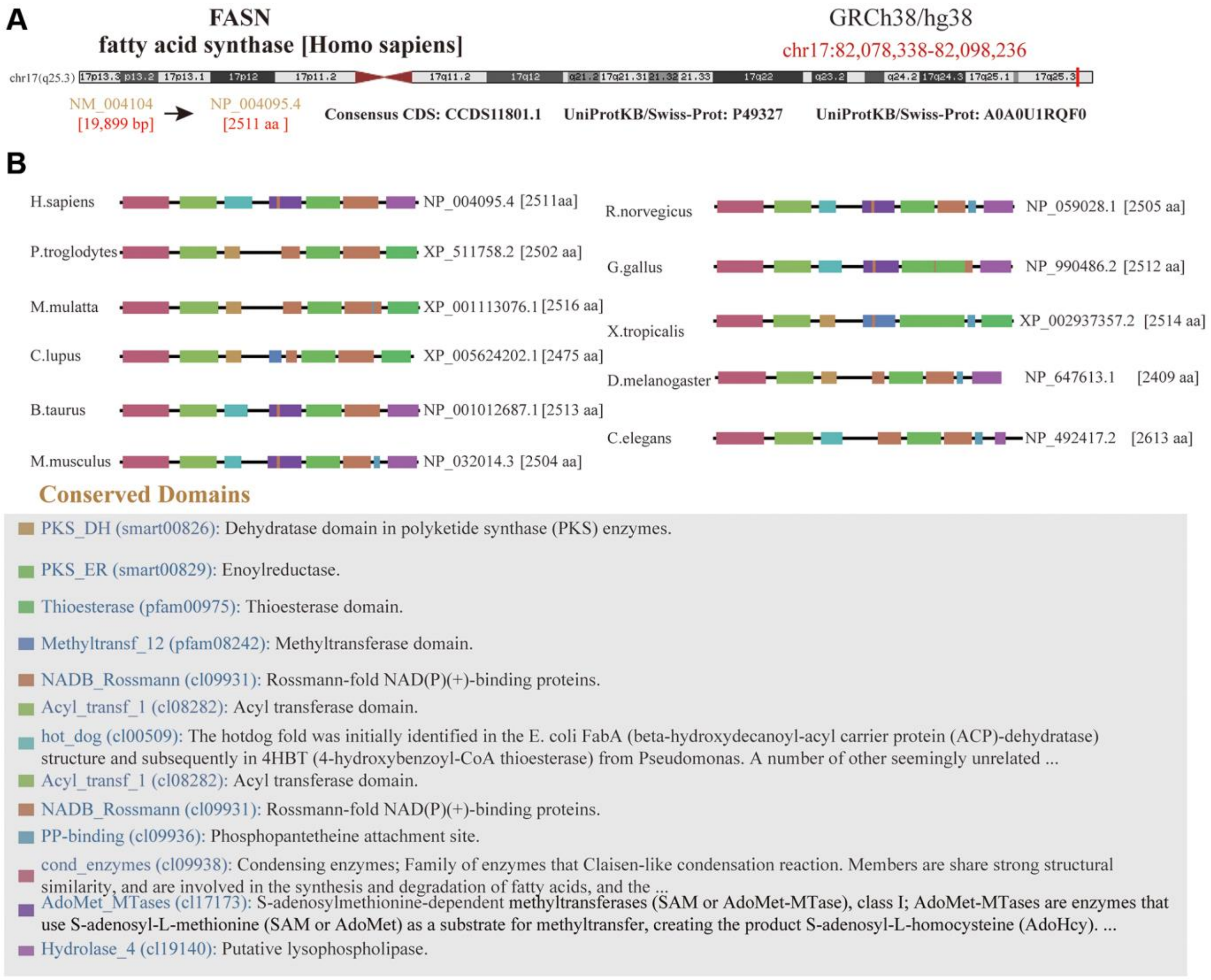

Supplementary Figure 1. Structural characteristics of FASN across various species. (**A**) Genomic location of FASN; (**B**) Conserved domains of the FASN protein.

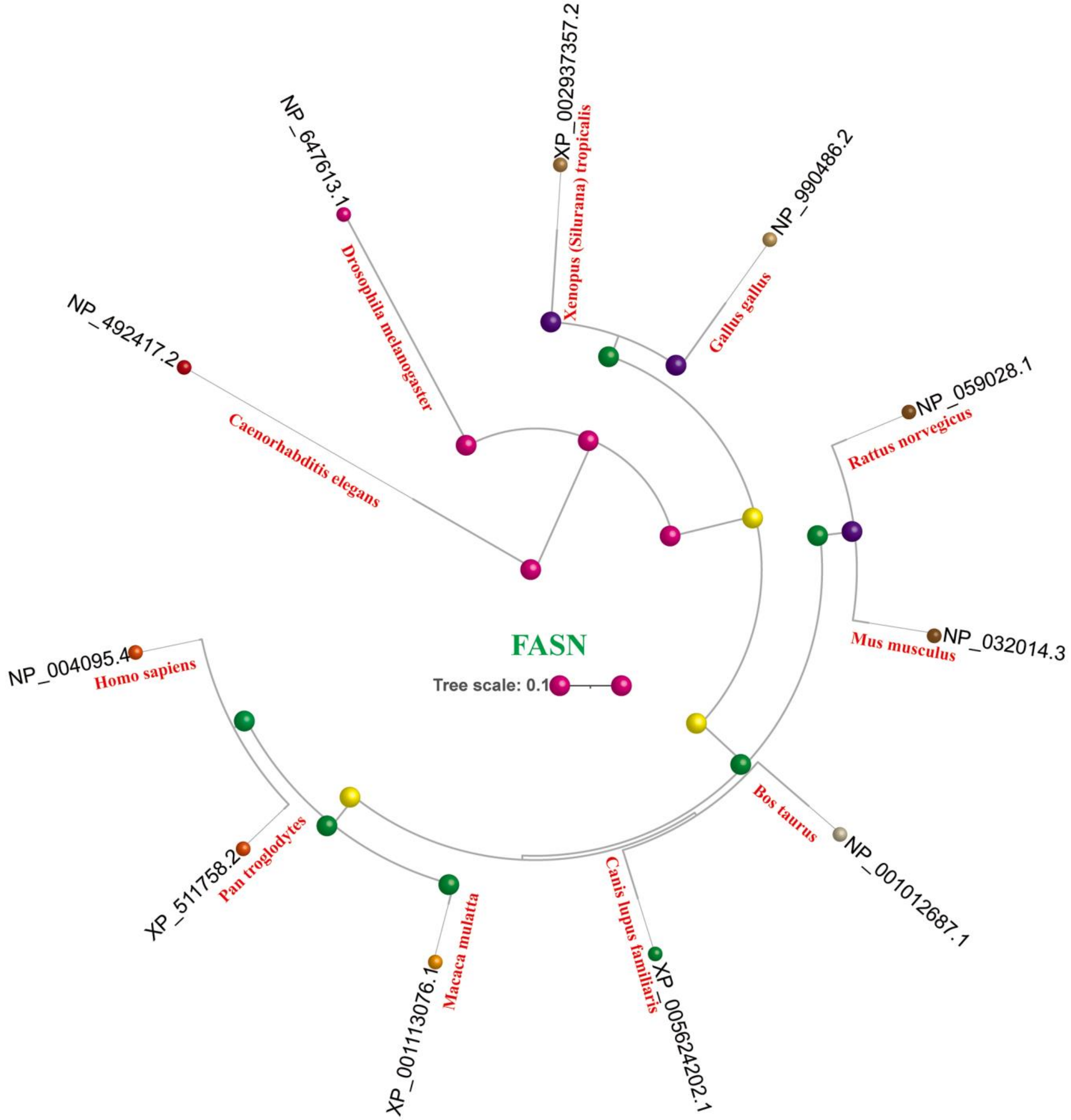

Supplementary Figure 2. Phylogenetic tree of FASN.
