# Supplementary material for: Landscape of the oncogenic role of fatty acid synthase in human tumors
Source: Aging (Albany NY). 2021 Dec 8;13(23):25106–37. doi: 10.18632/aging.203730 (PMC8714155; doi:10.18632/aging.203730)
Supplement: Supplementary Tables 1-6 [file aging-13-203730-s002.pdf]

SUPPLEMENTARY TABLES

Supplementary Table 1. Correlation of FASN and the outcome of breast cancer in subgroup analysis.

Factor	Subgroup	Sample size	OS		RFS		DMFS		PPS	
			HR	*P*	HR	*P*	HR	*P*	HR	*P*
**ER status**	ER positive	3499	1.22	0.2314	0.89	0.1831	1.14	0.3469	1.14	0.3469
	ER negative	2168	0.61	**0.0279**	0.84	0.0802	0.72	**0.0403**	0.72	**0.0403**
**TP53 status**	mutated	272	0.68	0.2892	1.55	0.075	0.56	0.2083	0.56	0.2083
	Wild type	388	1.6	0.2105	2.11	**0.0058**	2.65	**0.0192**	2.65	**0.0192**
**PR status**	PR positive	1559	2.27	0.0673	0.84	0.3087	0.81	**0.3891**	0.81	**0.3891**
	PR negative	1989	1.39	0.1936	0.81	0.0793	1.4	0.0621	1.4	0.0621
**HER2 status**	HER2 positive	1273	0.67	**0.0299**	0.78	0.053	0.77	0.1802	0.77	0.1802
	HER2 negative	6262	0.73	**0.0058**	0.71	**7.1e-09**	0.63	**2.9e-07**	0.63	**2.9e-07**
**Grade**	Grade 1	576	0.38	**0.0394**	0.39	**0.0103**	0.57	0.1728	0.57	0.1728
	Grade 2	1795	1.57	0.0543	1.14	0.3026	0.77	0.0714	0.77	0.0714
	Grade 3	2058	0.74	0.0549	0.83	0.0503	0.71	**0.0096**	0.71	**0.0096**
**Intrinsic subtype**	Basal	1494	0.81	0.2772	0.57	**4.7e-07**	0.78	0.1367	0.78	0.1367
	Luminal A	3511	0.77	0.1388	0.79	**0.0108**	0.82	0.1539	0.82	0.1539
	Luminal B	2015	0.82	0.3014	0.75	**0.0091**	0.69	**0.0094**	0.69	**0.0094**
	HER2+	515	0.37	**0.0005**	0.7	**0.0497**	0.64	0.0777	0.64	0.0777
**Lymph node status**	Lymph node positive	2153	0.61	**0.0037**	0.69	**1.1e-05**	0.55	**2.5e-06**	0.55	**2.5e-06**
	Lymph node negative	2829	0.69	0.0596	0.85	0.0779	0.65	**0.0016**	0.65	**0.0016**
**Pietenpol subtype**	Basal-like 1	418	1.65	0.2114	0.52	**0.0047**	1.69	0.096	1.69	0.096
	Basal-like 2	165	5.76	**0.0002**	1.64	0.1821	1.93	0.0823	1.93	0.0823
	immunomodulatory	462	0.54	0.1878	0.6	0.0434	0.46	**0.0304**	0.46	**0.0304**
	Mesenchymal	382	0.39	**0.0084**	0.46	**0.0001**	0.51	**0.0281**	0.51	**0.0281**
	Mesenchymal stem-like	201	0.27	**0.0074**	0.41	**0.0125**	0.37	0.0502	0.37	0.0502
	Luminal androgen receptor	413	0.4	**0.002**	0.56	**0.0033**	0.66	**0.1525**	0.66	**0.1525**

Abbreviations: HR: hazard ratio; OS: overall survival; RFS: relapse free survival; DMFS: distant metastasis free survival; ER: Estrogen receptor; PR: Progesterone receptor; HER2: human epidermal growth factor receptor-2; TP53: Tumor Protein P53; NA: not available data. *P* value less than 0.05 is shown in bold.

Supplementary Table 2. Correlation of FASN and the outcome of lung cancer in subgroup analysis.

Factor	Subgroup	Sample size	OS		FP		PPS	
			HR	*P*	HR	*p*	*HR*	*p*
**Histology**	adenocarcinoma	865	2.14	**9.4e-09**	1.49	**0.0129**	1.84	**0.0097**
	squamous cell carcinoma	675	1.2	0.1415	1.38	0.2151	0.35	0.0645
**gender**	female	817	2.54	**5.8e-09**	2.01	**7.8e-05**	1.77	**0.0064**
	male	1387	1.29	**0.0013**	1.7	**7.2e-05**	1.45	**0.0463**
**smoking history**	exclude those never smoked	970	1.74	**3.0e-05**	1.55	**0.0004**	1.16	0.3048
	only those never smoked	247	3.44	**2.3e-05**	2.05	**0.0026**	2.6	**0.0031**
**stage**	stage I	652	3.16	**0.0157**	0.34	**0.0046**	3.22	**0.0358**
	stage II	320	2.47	0.1393	3.46	**0.009**	NA	NA
**grade**	grade II	310	2.52	**0.0107**	0.23	0.0613	NA	NA
**AJCC stage t**	t1	475	1.26	0.1213	3.12	0.0841	NA	NA
	t2	686	0.52	0.1526	0.67	0.3663	0.69	**0.5304**
**AJCC stage n**	n0	863	0.39	**0.0396**	1.99	0.1257	1.89	0.4025
**AJCC stage m**	m0	818	0.46	0.1932	0.23	**0.0117**	NA	NA
**surgery**	only surgical margins negative	730	3.84	**0.0001**	2.93	**9.4e-05**	2.78	**0.0078**
**radiotherapy**	no	276	0.39	0.1143	0.48	0.2646	NA	NA
**chemotherapy**	no	317	1.52	0.4613	2.26	0.2962	NA	NA

Abbreviations: HR: hazard ratio; AJCC: American Joint Committee on Cancer; OS: overall survival; FP: first progression; PPS: post progression survival; NA: not available data. *P* value less than 0.05 is shown in bold.

Supplementary Table 3. Correlation of FASN and the outcome of ovarian cancer in subgroup analysis.

Factor	Subgroup	Sample size	OS		PFS		PPS	
			HR	*p*	HR	*p*	HR	*p*
**Histology**	Endometrioid	62	3.28	0.1677	3.89	0.1548	NA	NA
	Serous	1232	1.13	0.1439	1.29	**0.0014**	1.31	**0.0043**
**Stage**	Stage 1	107	1.89	0.2613	2.28	0.1184	NA	NA
	Stage 2	72	1.88	**0.2354**	3.01	**0.0326**	2.69	0.0923
	Stage 3	1079	0.88	**0.122**	1.22	**0.0161**	1.22	**0.048**
	Stage 4	189	1.57	**0.018**	1.33	0.1413	1.39	0.1421
**Grade**	Grade 1	56	1.52	0.3954	0.44	0.1315	NA	NA
	Grade 2	325	0.78	0.1418	1.4	0.0592	1.33	0.1478
	Grade 3	1024	0.87	0.0895	1.17	0.0627	1.23	**0.0597**
	Grade 4	21	0.59	0.3614	NA	NA	NA	NA
**TP53 mutation**	Mutated	516	1.4	**0.0036**	1.57	**0.0001**	1.53	**0.0008**
	Wild type	102	1.84	**0.0272**	0.71	0.2007	2.05	**0.0193**
**Debulk**	optimal	802	0.64	**1.5e-05**	0.74	**0.0023**	0.7	**0.0064**
	suboptimal	536	0.83	0.1182	0.85	0.1764	1.43	**0.0077**
**Chemotherapy**	Contains platin	1438	0.92	**0.2755**	0.86	**0.0343**	1.28	**0.0056**
	Contains Taxol	821	0.78	**0.0105**	0.79	**0.0108**	1.17	0.1722
	Contains Taxol+platin	804	0.8	**0.021**	0.82	**0.0314**	1.17	0.1647
	Contains Avastin	50	0.64	0.3165	1.54	0.2075	0.62	0.286
	Contains Docetaxel	108	1.47	0.233	1.53	0.0853	1.57	0.2054
	Contains Gemcitabine	135	0.73	0.1477	0.73	0.1155	0.67	0.0739
	Contains Paclitaxel	248	1.5	0.0856	1.58	**0.0076**	1.68	0.0506
	Contains Topotecan	119	0.56	**0.0097**	1.26	0.2572	0.56	**0.0068**

Abbreviations: HR: hazard ratio; OS: overall survival; PFS: progress free survival; PPS: post progression survival; TP53: Tumor Protein P53; NA: not available data. *P* value less than 0.05 is shown in bold.

Supplementary Table 4. Correlation of FASN and outcome of gastric cancer in subgroup analysis.

Factor	Subgroup	Sample size	OS		FP		PPS	
			HR	*P*	HR	*P*	HR	*P*
**Gender**	Female	244	2.2	**1.0e-05**	2.06	**0.0001**	2.67	**2.3e-06**
	Male	566	2.34	**2.1e-12**	2.18	**9.8e-11**	3.25	**< 1e-16**
**Stage**	Stage 1	69	3.41	**0.0263**	2.33	**0.1551**	4.08	**0.0474**
	Stage 2	145	1.85	0.0506	1.63	0.1271	1.83	0.067
	Stage 3	319	2.26	**1.3e-06**	1.94	**0.0008**	2.75	**2.6e-06**
	Stage 4	152	1.57	**0.023**	1.54	**0.0349**	1.82	**0.0135**
**Stage t**	t2	253	1.87	**0.0044**	1.78	**0.006**	2.08	**0.0016**
	t3	208	1.76	**0.0014**	1.6	**0.0128**	2.42	**4.8e-06**
	t4	39	0.72	0.4482	1.37	0.4223	0.48	0.1121
**Stage n**	n0	76	1.81	0.1747	1.76	0.1894	0.55	0.3446
	n1	232	2.13	**0.0002**	2.15	**0.0008**	3.3	**2.3e-07**
	n2	129	2.46	**7.0e-05**	2.01	**0.0014**	2.62	**6.5e-05**
	n3	76	1.54	0.1166	1.44	0.1824	1.56	0.146
**Stage m**	m0	459	1.72	**0.0001**	1.53	**0.0015**	2.38	**6.9e-09**
	m1	58	1.78	**0.0493**	1.37	0.2871	3.15	**0.0037**
**HER2**	negative	641	1.92	**1.6e-08**	1.95	**6.9e-07**	2.56	**1.9e-10**
	positive	424	1.89	**3.9e-05**	2.59	**6.6e-09**	5.07	**3.1e-11**
**Lauren classification**	Intestinal	336	2.53	**5.3e-09**	1.94	**0.0004**	3.87	**2.6e-11**
	Diffuse	248	1.76	**0.0029**	1.61	**0.0115**	1.61	**0.0195**
	Mixed	33	0.36	0.1051	0.44	0.1198	NA	NA
**Differentiation**	Poorly	166	1.36	0.1344	1.34	0.2102	1.35	0.3727
	Moderately	67	1.64	0.1359	1.42	0.3195	4.23	**0.0032**
	Well	32	2.06	0.1872	NA	NA	NA	NA
**Treatment**	Surgery alone	393	1.43	**0.0162**	1.25	0.1168	2.24	**1.4e-06**
	5-Fu based adjuvant	157	1.88	**0.001**	1.97	**0.0001**	1.24	0.238
**Perforation**	No	169	1.15	0.4961	1.2	0.3664	1.74	0.0633

Abbreviations: HR: hazard ratio; OS: overall survival; FP: first progression; PPS: post progression survival; HER2: human epidermal growth factor receptor-2; NA: not available data. *P* value less than 0.05 is shown in bold.

Supplementary Table 5. Correlation of FASN and outcome of liver cancer in subgroup analysis.

Factor	Subgroup	Sample size	OS		PFS		RFS		DSS	
			HR	*P*	HR	*P*	HR	*P*	HR	*P*
**Stage**	Stage 1	171	1.56	0.2807	0.73	0.2046	0.62	0.0792	0.34	0.0685
	Stage 2	86	2.73	0.0562	2.15	**0.0134**	2.3	**0.0164**	2.35	0.1307
	Stage 3	85	0.53	0.0511	1.54	0.1182	1.71	0.0763	0.58	0.1695
**Grade**	Grade 1	55	0.56	0.2875	0.63	0.2346	0.33	**0.0199**	0.5	0.3097
	Grade 2	177	2.01	**0.0105**	1.77	**0.0182**	1.8	**0.0205**	2.58	**0.0053**
	Grade 3	122	1.41	0.2612	1.74	**0.0297**	1.64	0.0713	1.43	0.347
**AJCC_T**	T1	181	1.51	0.2861	0.79	0.3241	0.67	0.1283	0.62	0.3015
	T2	94	1.99	0.0654	2.09	**0.0106**	2.4	**0.0073**	2.03	0.1478
	T3	80	0.39	**0.0036**	1.53	0.1383	1.68	0.0998	0.4	**0.0204**
**Gender**	Female	121	1.72	0.059	1.48	0.165	1.39	0.3534	1.57	0.3201
	Male	250	0.78	0.2945	1.34	0.1135	1.39	0.1314	1.4	0.2392
**Vascular invasion**	None	205	0.8	0.4055	1.35	0.2039	1.29	0.3093	0.73	0.3937
	micro	93	1.95	0.1742	0.56	**0.045**	0.58	0.0935	0.32	**0.0487**
**Race**	White	184	0.65	0.0851	0.7	0.0699	0.69	0.1061	0.69	0.2204
	Asian	158	2.13	0.0605	1.73	**0.0228**	1.83	**0.0183**	2.22	0.133
**Sorafenib treatment**	treated	30	1.97	0.2196	0.49	0.101	0.57	0.2987	1.97	0.2196
**Alcohol consumption**	Yes	117	1.98	0.1211	2.03	**0.0386**	1.78	0.0651	2.05	0.0562
	none	205	1.31	0.3425	0.63	**0.0249**	0.6	**0.0245**	0.55	0.0612
**Hepatitis virus**	Yes	153	0.49	0.0806	1.3	0.2643	1.35	0.2444	0.44	0.1206
	none	169	1.59	**0.0482**	1.41	0.1555	0.78	0.3237	1.69	0.0771

Abbreviations: HR: hazard ratio; AJCC: American Joint Committee on Cancer; OS: overall survival; PFS: progress free survival; RFS: relapse free survival; DSS: disease specific survival; NA: not available data. *P* value less than 0.05 is shown in bold.

Supplementary Table 6. Phosphorylation locus of FASN.

site	sequence	experimentally confirmed#	hydrophobicity	*p*-site similarity score	maximum kinase specificity	sum kinase specificity score	conservation score
**S207**	FLRLGMLSPEGTCKA	24719451; 25850435	0.253	–57.6	516	20,583	42.7
**S724**	IPEAQWHSSLARTSS	20068231	–0.827	–59.2	273	12,141	30.9
**S725**	PEAQWHSLARTSSA	20068231	–1.007	–57.5	353	15,253	16.4
**T976**	FDHPESPTPNPTEPL	18669648	–1.440	–54.8	278	9,657	9.7
**S1174**	AQIPRDPSQQELPRL	27251275	–1.267	–55.3	572	20,371	4.6
**S1411**	RRPTPQDSPIFLPVD	18669648	–0.860	–56.2	549	21,758	10.6
**S2198**	SSKADEASELACPTP	18669648	–0.760	–55.9	289	13,190	1.5
**T2204**	ASELACPTPKEDGLA	15302935	–0.320	–55.5	283	9,599	14.2

#The PMID (PubMed Unique Identifier) information of the publication was provided.
